# Biomolecular glass with amino acid and peptide nanoarchitectonics

**DOI:** 10.1126/sciadv.add8105

**Published:** 2023-03-17

**Authors:** Ruirui Xing, Chengqian Yuan, Wei Fan, Xiaokang Ren, Xuehai Yan

**Affiliations:** ^1^ State Key Laboratory of Biochemical Engineering, Institute of Process Engineering, Chinese Academy of Sciences, Beijing 100190, China.; ^2^ School of Chemical Engineering, University of Chinese Academy of Sciences, Beijing 100049, China.; ^3^ Center for Mesoscience, Institute of Process Engineering, Chinese Academy of Sciences, Beijing 100190, China.

## Abstract

Glass is ubiquitous in life and widely used in various fields. However, there is an urgent need to develop biodegradable and biorecyclable glasses that have a minimal environmental footprint toward a sustainable society and a circular materials economy. Here, we report a family of eco-friendly glasses of biological origin fabricated using biologically derived amino acids or peptides through the classic heating-quenching procedure. Amino acids and peptides with chemical modification at their ends are found able to form a supercooled liquid before decomposition and eventually glass upon quenching. These developed glasses exhibit excellent glass-forming ability and optical characteristics and are amenable to three-dimensional–printed additive manufacturing and mold casting. Crucially, the glasses show biocompatibility, biodegradability, and biorecyclability beyond the currently used commercial glasses and plastic materials.

## Teaser

Biodegradable and biorecyclable glasses developed from amino acids exhibit functionality and sustainability.

## INTRODUCTION

The materials and chemicals for tomorrow’s earth are highly hoped to be benign rather than toxic, renewable rather than depleting, and degradable rather than persistent (*1*). Glass is one of the most important high-performance materials in human civilization (*2*, *3*). A variety of glass-forming components have been developed as structural building units for the preparation of glasses. For example, the most well-known inorganic glass is usually manufactured from sand, lime, and sodium carbonate (*4*); poly(methyl methacrylate) (PMMA) glass is a transparent and rigid thermoplastic organic material (*5*); metallic glass (or glassy metal) is a noncrystalline material composed of either pure metal or a combination of metal and metalloid components (*6*); the recently reported metal-organic framework glass is usually composed of metal ions or clusters coordinated by organic ligands arranged in tetrahedral units (*7*). However, these glasses are biologically incompatible and not readily degraded in nature, which will cause long-term ecological and environmental burdens for the green life future (*1*, *8*, *9*). Therefore, the development of biodegradable and biorecyclable glasses is expected to have a minimal environmental footprint and to contribute to a lasting improvement among ecological and social impacts.

Amino acids and peptides are abundant biomolecules in living organisms and are widely used for the development of nanoarchitectured materials with functional performances (*10*, *11*). The materials with amino acid or peptide nanoarchitectonics are considered completely eco-friendly because they can be reused in ecological system (*12*–*15*). Currently, the predominant approach to making glass is to heat glass formers at a high temperature (above their melting temperature, *T*_m_) to form a supercooled liquid and then to quench this liquid by cooling at a sufficient fast rate to prevent crystallization (*4*). Glass is a nonequilibrium, noncrystalline state of matter that lacks the periodicity of crystals but behaves mechanically like a solid, depending upon the thermal history of the melt (*2*, *16*, *17*). However, native amino acids and peptides have poor thermal stability and easily decompose into amines and carbon dioxide (CO_2_) at high temperatures at or near *T*_m_ (*18*). As a result, glass cannot be processed and obtained using these native biomolecules. It is reported that amino acids and peptides when subjected to chemical modification at the ends by hydrophobic groups can notably improve their thermal stability (*19*), which inspired us that it is possible to use the modified amino acids and peptides to process glass.

**Fig. 1.**
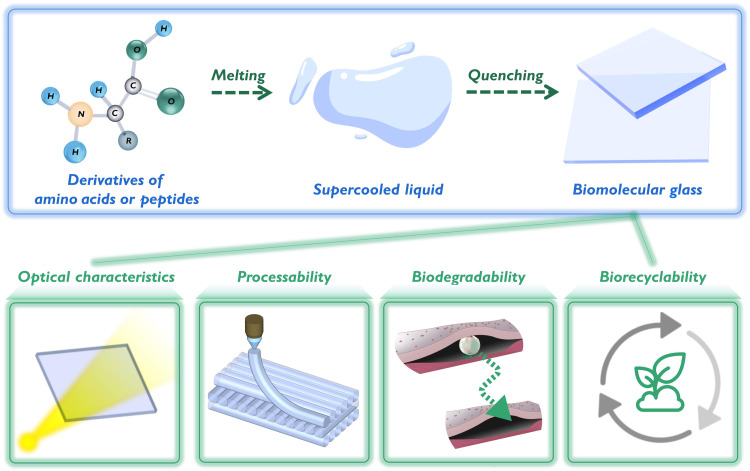
Schematic diagram of the biomolecular glass. The structural units are derivatives of molecular amino acids or peptides used to prepare a supercooled liquid through a high-temperature melting process and then processed into a glass by a quenching procedure. These glasses had excellent optical characteristics, flexible processability, as well as biodegradability and biorecyclability.

In this work, we used chemically modified amino acids and peptides as the glass formers to successfully fabricate biomolecular glasses with biodegradability and biorecyclability (Fig. 1). We melted them to first form a supercooled liquid upon heating in an inert gas atmosphere before reaching their decomposition temperature (*T*_d_). Through the accurate control of the heating and cooling rates, the supercooled liquid was quenched to form glass finally, and crystallization was effectively prevented. We evaluated the glass-forming ability (GFA) and glass performance of representative biomolecular glasses derived from amino acids or peptides. Kinetic and thermodynamic parameters related to the glass transition were determined. Subsequently, we studied the ability of the glasses to transmit light and their adaptability for additive manufacturing [three-dimensional (3D) printing and mold casting]. To determine whether the developed biomolecular glass was eco-friendly, we conducted biodegradation experiments in vitro and in vivo as well as composting experiments. We found that the biomolecular glasses based on derivatives of amino acids or peptides had a unique combination of functional properties and eco-friendly features, excellent optical characteristics, good mechanical properties, and flexible processability, as well as the desired biodegradability and biorecyclability (*20*). It is worth emphasizing that these biomolecular glasses are completely different from bioactive glasses (BAGs)/bioglasses, which were discovered in 1969 by Hench (*21*, *22*). BAG is a kind of bioactive ceramic material that exhibits bone regeneration properties with a composition containing more calcium and phosphate than silica glasses, and belonged to the SiO_2_-Na_2_O-CaO-P_2_O_5_ inorganic silicate glass system. Whereas these biomolecular glasses are far from large-scale commercialization, there is an interest in the development of glasses with eco-friendly performance, which may be a giant leap toward a sustainable future.

## RESULTS

### Glass preparation and characterization

To enable the formation and subsequent quenching of supercooled liquids without molecular decomposition, we chemically modified a series of amino acids and peptides using acetyl (Ac-), 9-fluorenylmethyloxycarbonyl (Fmoc-), and benzyloxycarbonyl (Cbz-) groups. Upon heating, randomly selected native amino acids, including glutamine (Q), histidine (H), phenylalanine (F), and tyrosine (Y), were found to undergo a total weight loss greater than 15%, whereas valine (V) and leucine (L) burned completely, with a total weight loss greater than 90% at *T*_m_ (fig. S1). It was found that the *T*_m_ values of these modified molecules were far below their decomposition temperatures (table S1). Next, biomolecular glasses were successfully prepared from these modified amino acids and peptides by following programmed heating and quenching procedures at heating and cooling rates of 10 K min^−1^ in an inert atmosphere (fig. S2 and table S2). The successful melting of the biomolecular compounds before decomposition is undoubtedly a breakthrough for the preparation of biomolecular glasses.

**Fig. 2.**
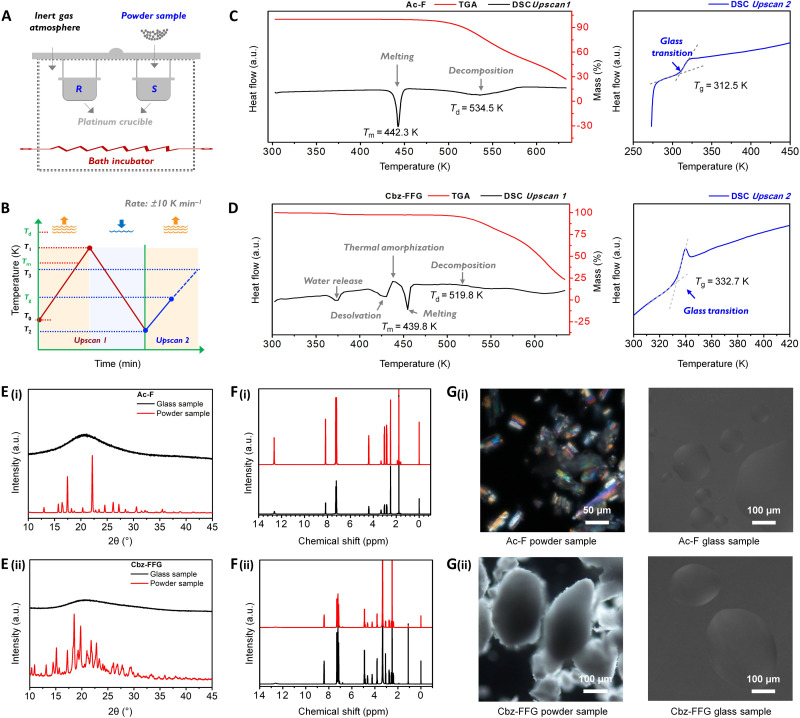
Glass transitions and GFA of amino acids and peptides. (**A**) Schematic diagram of the DSC-TGA equipment. *R* represents the reference crucible, and *S* denotes the sample crucible. (**B**) Procedure for the DSC-TGA measurements. For the *Upscan* 1 stage, the powder sample was heated from room temperature (*T*_0_) to a temperature (*T*_1_) greater than *T*_m_ but less than *T*_d_. Quenching treatment followed an isothermal procedure at *T*_1_ for 15 min. For the *Upscan* 2 stage, the product was reheated from *T*_2_ to *T*_3_, and the glass transition was observed. Thermogravimetric analysis and enthalpic responses of the Ac-F glass (**C**) and Cbz-FFG glass (**D**). *T*_m_, *T*_d_, water release, desolvation, and thermal amorphization are indicated in the *Upscan* 1 curves. *T*_g_ is indicated in the *Upscan* 2 curves. The heating and cooling rates were ±10 K min^−1^. The XRD patterns (**E**), ^1^H solution NMR spectra (**F**), and POM images (**G**) of Ac-F glass (i) and Cbz-FFG glass (ii) show the obvious crystalline nature of the raw material powders and the amorphous nature of the glasses; the chemical structure of the molecules did not change obviously during the formation of the glass. a.u., arbitrary units.

The formation of glass from amino acids or peptides can be monitored by changes in enthalpy or volume as a function of temperature. Here, an Ac-modified amino acid (Ac-F) and a Cbz-modified tripeptide [Cbz-(D)-FF-glycine (Cbz-FFG)] were selected as representative samples. Thermal gravimetric analysis (TGA) and differential scanning calorimetry (DSC) measurements were performed under nitrogen (Fig. 2A) according to a preset temperature program (Fig. 2B). In the TGA curve and enthalpic response of Ac-F obtained in the *Upscan* 1 region, we observed a sharp endothermic peak at *T*_m_ = 442.30 K followed by a broad decomposition peak centered at *T*_d_ = 534.50 K (Fig. 2C). The DSC-TGA results showed no evidence of qualitative decomposition or volatilization during the formation of the glass. Upon reheating the amorphous product, a typical glass transition was observed. Glass transition temperature (*T*_g_) was measured as the onset temperature of the glass transition peak, as the temperature at the intercept between an extrapolated straight line of the enthalpy curve and a tangent line at the inflection point of the sharply rising enthalpy curve (*23*). In the enthalpic responses of Ac-F obtained in the *Upscan* 2 region, we observed the glass transition at *T*_g_ = 312.50 K. The increase in the glass transition heat capacity (∆*C*_p_) for the transition from a glass to a supercooled liquid at *T*_g_ was calculated to be 0.70 J K^−1^ g^−1^. Next, similar experiments were performed for the tripeptide derivative Cbz-FFG. In the TGA curve for this sample, peaks were observed at 374.60, 428.20, 438.60, and 453.30 K, corresponding to water release, solvent loss, exothermic thermal amorphization, and melting, respectively (Fig. 2D). During this process, a weight loss of 3.86% was observed before peptide decomposition. *T*_g_ was determined to be 332.70 K, and ∆*C*_p_ was calculated to be 0.67 J K^−1^ g^−1^. These results clearly put in evidence that temperature-driven glass transitions can be achieved by modified amino acids or peptides.

The x-ray diffraction (XRD) patterns of the Ac-F and Cbz-FFG powders showed sharp diffraction peaks, which indicated that the raw powders had a crystalline nature, suggesting that long-range order occurred within the powder samples (Fig. 2E and fig. S3). In contrast, broad bands without well-defined diffraction peaks were observed in the XRD patterns of their glassy counterparts, reflecting an amorphous nature of the glass samples (*24*). Polarized optical microscopy (POM) was then performed to determine the phases of the crystalline and glassy states (Fig. 2G). For the Ac-F and Cbz-FFG powder samples, strong optical anisotropy was observed, indicating the orientational organization within the crystalline powders. In sharp contrast, no detectable anisotropic birefringence was observed for either the Ac-F or Cbz-FFG glasses, indicating that these samples had an isotropic and amorphous feature. Notably, the ^1^H solution nuclear magnetic resonance (NMR) spectra (Fig. 2F) confirmed that there were no changes in the chemical structures of these glass samples during the melting and quenching procedures. These results indicated that the modified amino acids and peptides are robust for formation of glass through the melting and quenching approach.

However, it is worth mentioning that not all obtained glasses are colorless and transparent (table S2), which is related to the structural and physicochemical properties of the amino acid derivatives. For example, the glasses formed by derivatives of amino acid tryptophan (W) showed distinct yellow color. The emergence of exothermic peaks with slight weight increase in DSC-TGA curves before the *T*_m_ indicated the occurrence of oxidation event of Fmoc-W and Cbz-W during the heating process (fig. S4). Furthermore, liquid chromatography coupled with high-resolution electrospray ionization mass spectrometry (LCMS) was used to quantify the chemical changes of these derivatives before and after glass formation. Integral area loss of ingredients [mass/charge ratio (*m/z*) of Fmoc-W = 427.1650, *m/z* of Cbz-W = 339.1339] was calculated as 5.23 and 2.96%, respectively (figs. S5 and S6), which can be attributed to the oxidation and oxidative decomposition caused by high temperature. Besides, the DSC-TGA result of Ac-protected asparagine (Ac-N) showed no evidence of qualitative decomposition or oxidation during the glass formation (fig. S7). In addition, no obvious signals were captured for the decomposition or oxidation of Ac-N before and after glass formation from LCMS (fig. S8). Amino acid derivatives containing arginine (R) could not be prepared to form glasses through the melting-quenching strategy. Taking Cbz-R as an example, we found that it could not form a supercooled liquid upon heating before reaching its *T*_d_ and severe decomposition occurred at *T*_m_, which were also confirmed by DSC-TGA (fig. S9) and LCMS (figs. S10 and S11).

### Glass transition mechanism and computational simulations

**Fig. 3.**
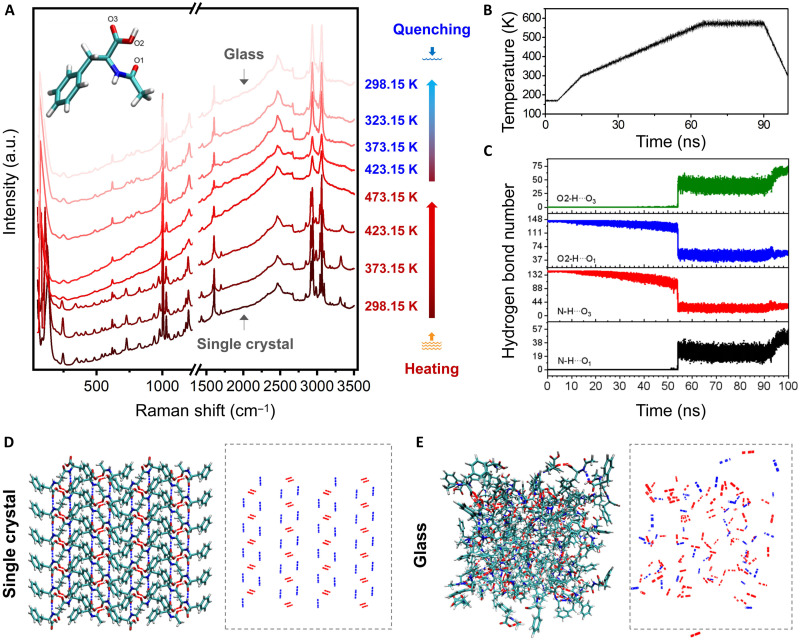
Molecular mechanism of the formation of a glass starting from a single crystal. (**A**) In situ Raman spectra of Ac-F as a function of temperature. The Ac-F single crystal was heated, melted, and then quenched to form a glass. The inset shows the chemical structure of Ac-F with the oxygen atom types labeled. (**B**) Temperature change curve during the simulated annealing process. (**C**) Evolution of the number and types of hydrogen bonds during the simulated annealing process. Molecular packing modes within Ac-F single crystal (**D**) and glass obtained through molecular dynamics simulations (**E**), indicating regularly arranged hydrogen bond networks in the crystalline state and a chaotic molecular arrangement in the glassy state.

To probe the mechanism of glass transition, variable-temperature in situ XRD and Raman spectroscopy were conducted over the entire phase transition process (from crystals to glass) of Ac-F. When the temperature was increased from 298.15 to 397.15 K, no obvious changes in the XRD patterns were observed (fig. S12), suggesting a stable ordered molecular organization in the starting crystals. This stability was confirmed by the Raman spectra, evidenced by no signal change over the range from 298.15 to 423.15 K (Fig. 3A). When the temperature was further increased above *T*_m_ = 442.30 K, the sharp Bragg peaks in the XRD pattern disappeared, implying the collapse of the long-range order in the crystalline structures. This can be attributed to the disruption of the regular molecular arrangement, which was confirmed by the disappearance of the fine peaks in the Raman spectra, especially the C═O stretching band of the carboxyl group at 1730 cm^−1^, the O─H stretching band near about 3400 cm^−1^, the N─H stretching band in the range from 3324 to 3200 cm^−1^, and the C─H stretching band occurring at 3100 to 2800 cm^−1^ (*25*). During the quenching process, no sharp Bragg peaks were observed, suggesting that quenching prevented the Ac-F supercooled liquid from following the thermodynamically favorable path toward crystalline formation and trapped the system in a metastable amorphous state. In addition, small increases in the intensity of the N─H and O─H stretching bands in the Raman spectra were observed, suggesting that molecular rearrangement occurred during the quenching process (*26*). The Raman bands corresponding to the C═O stretching, the N─H stretching, and the C─H stretching vibrations and the ^13^C signals in the solid-state NMR spectra (ssNMR) (fig. S13) were obviously broadened, demonstrating the coexistence of multiple types of hydrogen bonding interactions in the glass (*27*, *28*). Similar evidence of molecular rearrangements was observed during the formation of melt-quenched (MQ) Cbz-FFG glass (fig. S14). In addition, we repeated the formation process of Cbz-FFG glass and recorded the in situ Raman spectra of Cbz-FFG as a function of temperature in successive “heating-cooling” cycles. The results indicated that the above rules of molecular arrangements at each stage of glass formation (including heating, melting, and cooling) were not changed (fig. S15). LCMS was used to separate and identify the components of Cbz-FFG glasses, which have undergone one to three cycles of reproducing. These glasses were essentially formed by the same constituents and proportions. Compared with the glass former (Cbz-FFG), no oxidation and decomposition products were found in glasses (figs. S16 and S17). Together, these results excluded the decomposition of Cbz-FFG molecules and ensured the reproducibility of Cbz-FFG peptide glass.

Furthermore, to investigate the molecular mechanism underlying the vitrification of amino acids and peptides, a combination of ssNMR, in situ Raman spectroscopy, and all-atom molecular dynamics (AAMD) simulations was performed (Fig. 3). Ac-F was selected as a simple typical model to elucidate the evolution of molecular packing patterns from single crystal (table S3) to glass. The analysis on single-crystal structure of Ac-F revealed that two kinds of predominant hydrogen bonds, ─N─H···O═C (N─H···O_3_) and ─O─H···O═C (O_2_─H···O_1_), stabilized the single crystal (Fig. 2A, inset, and D, and fig. S18A) (*29*), which was consistent with the sharp peaks in the Raman spectra (Fig. 2A) and ssNMR spectra (fig. S13). Upon heating (Fig. 3B), the abundance of these two types of hydrogen bonds decreased, indicating loss of the single-crystal structure (Fig. 3C). After the subsequent melting-cooling treatment, multiple types of hydrogen bonds, especially O_2_─H···O_3_ and N─H···O_1_, appeared and became more abundant (Fig. 3E and fig. S18B), consistent with the emergence of broadened bands in the Raman and ssNMR spectra and implying the formation of an amorphous glass. If the cooling rate was slow enough, such as equal to or less than 0.5 K min^−1^, Ac-F crystals in crystalline state can be regained, but not single crystal through such a strategy (fig. S19). Therefore, it was concluded that the glass transition was a kinetic-freezing process (*30*) and that the cooperation of multiple weak intermolecular interactions facilitated the stabilization of the Ac-F glass with disordered molecular packing.

Since the glass transition is considered a kinetic phenomenon, the final enthalpy and *T*_g_ of the glass depend on the cooling rate (*31*). To confirm the above conclusion, the *T*_g_ values of glasses obtained at different cooling rates were investigated due to the dependence of *T*_g_ on the chemical structure and mobility of the glass formers. Figure S20 shows the enthalpy change curves of Ac-F as a function of temperature at different cooling rates (termed *q*_c_). The heating rate (termed *q*_h_) equaled *q*_c_ for all the DSC tests. These results showed that *T*_g_ decreased with increasing cooling rate and reached a plateau above 10 K min^−1^. At a higher cooling rate, lower *T*_g_ values were observed, suggesting that the system had less time for molecular rearrangement and resulted in looser molecular packing networks.

### GFA and the fragility index (*m*)

GFA refers to the ability of a liquid to avoid crystallization during quenching and is usually measured by reduced glass transition temperature (*T*_rg_, which is defined as *T*_g_/*T*_m_). Systems with higher *T*_rg_ values (>2/3) have greater thermal stability and GFA (*32*). The *T*_rg_ values were calculated to be 0.71 for Ac-F and 0.73 for Cbz-FFG (table S1), which satisfy the requirement of Kauzmann’s 2/3 law (*4*), indicating that Ac-F melts have good resistance to crystallization in their supercooled state. However, not all molecules conformed to this empirical rule. For example, although Ac-G and Ac-Q exhibited lower *T*_rg_ values of 0.61 and 0.64, respectively, they still formed transparent glasses (fig. S21 and table S1). The GFA is often influenced by competing effects that suppress local order, leading to crystal formation (*33*). Molecules with lower *T*_rg_ values usually show a strong crystallization tendency, and these components are not desired in glass manufacturing. Notably, Kauzmann’s 2/3 law originates from data on routine MQ glasses based on traditional ionic and covalent bonds (*34*), so the observation that the glasses developed in this study do not all obey this law suggests that these glasses based on amino acids and peptides represent a distinctive glass type.

The fragility index *m*, which corresponds to the activation energy of viscosity (η) at *T*_g_ (*32*), was calculated to be 48 for Ac-F glass and 63 for Cbz-FFG glass (table S1). These results are comparable to those for As_2_Se_3_ (*m* = 47) and B_2_O_3_ (*m* = 50) (*35*) and superior to those for PMMA (*m* = 145) (*36*). Strong supercooled liquids typically have low values of *m*, while fragile supercooled liquids have high values of *m* (*37*). The *m* values of phenylalanine derivatives showed distinct differences. For example, the Cbz-F glass exhibited a low fragility (*m* = 13), and the Fmoc-F glass exhibited a high fragility (*m* = 58). Likewise, the *m* value of Fmoc-G glass (*m* = 51) was greater than that of Ac-G glass (*m* = 15). These differences can be attributed to the structural rigidity of the Fmoc group. Another possible explanation could be related to the enhanced hydrophobic effects due to the presence of Fmoc groups. All of these suggest that modified amino acids or peptides have a good GFA and have potential to be processed into glass (figs. S21 to S24 and table S1).

### Additive 3D manufacturing and mold casting

**Fig. 4.**
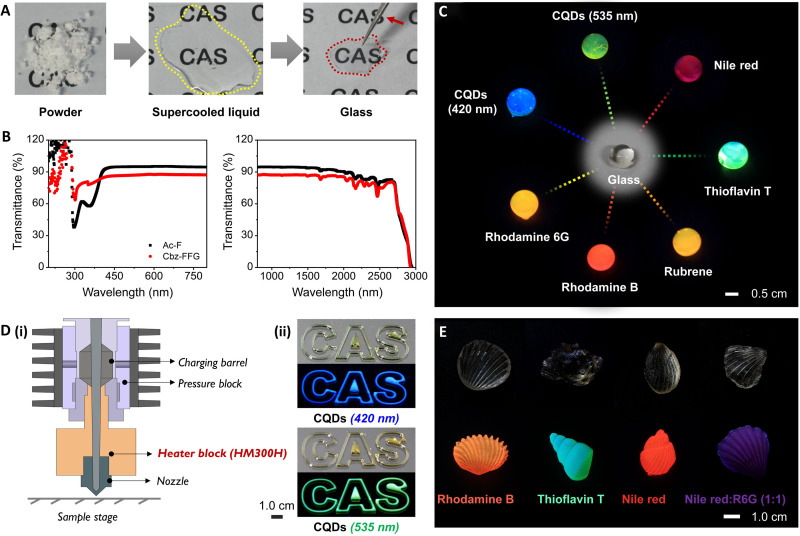
Light transmittance and additive manufacturing of glasses. (**A**) Photographs of the glass transition of Ac-F from the crystalline state to the glassy state during heating-cooling treatment. The red arrow indicates the tweezer. (**B**) Transmittance spectra of glasses in the UV-visible region (left) and NIR region (right). (**C**) Photographs of an Ac-F glass doped with CQDs or dyes that emitted diverse fluorescence upon excitation with UV light at a wavelength of 365 nm. The middle image shows the pure glass without doping by fluorescent species. (**D**) Additive manufacturing of Ac-F fluorescent glass using a 3D printer via a well-designed program. (i) Schematic diagram of the 3D printer with a temperature-controlled barrel; (ii) photographs of the printed glass architecture (top: bright field; bottom: fluorescence field). Note: The fluorescence results for the encapsulated CQDs. “CAS” is the abbreviation for the Chinese Academy of Sciences. (**E**) Photographs of dark field (top) and fluorescent (bottom) glasses cast with commercial molds. Note: The mass ratio of CQDs or dyes to biomolecules was 1:10^5^, and the powders were evenly mixed before melting.

Precision glass processing opens up an enormous variety of possible glass types and geometries (*38*). The glass transition process of the Ac-F sample is schematically demonstrated in Fig. 4A. An obvious phase transition was observed, with the structure changing from a crystalline state (raw material powder) to an intermediate state (transparent viscous liquid) and finally to a glassy state (transparent solid). To investigate the optical performance of these transparent glasses, transmittance spectra in the ultraviolet (UV)–visible (200 to 800 nm) and near-infrared (NIR) (800 to 3000 nm) regions were obtained (Fig. 4B). These developed glasses exhibited good optical performance (up to ~90% transmittance), which was superior to that of the glass used in ordinary commercial lighting applications (80% on average) (*39*). In addition, carbon quantum dots (CQDs) and dyes were mixed with transparent viscous liquids of the biomolecules, and a series of colored glasses with diverse fluorescence were prepared (Fig. 4C). The above results indicated that these glasses had high optical transmittance and flexible processability. Besides, no obvious quenching or shift of organic fluorophores caused by the interactions between aromatic glass components and organic fluorescent groups was observed. For example, after mixing with equivalent of rhodamine B, the fluorescence spectra of Ac-modified amino acid (Ac-P and Ac-L) glasses and Fmoc-modified amino acid (Fmoc-P and Fmoc-L) glasses remained almost unchanged (fig. S25).

Next, we prepared 3D-printed Ac-F glass samples showing the clear and stereoscopic logo of “CAS” (abbreviation for Chinese Academy of Sciences) using a commercial biological 3D printer with a temperature-controlled barrel (Fig. 4D). XRD pattern of the 3D-printed Ac-F glass showed broad bands without well-defined diffraction peaks, reflecting an amorphous nature of the glass sample (fig. S26). As a further test, we poured uniform and bubble-free supercooled liquid into specific molds and obtained a series of bionic marine shells without distortion (Fig. 4E). Normally, fused glasses are notoriously difficult to shape; glasses characterized by a high softening point usually show a strong crystallization tendency during processing (*40*). These drawbacks have made traditional glasses inaccessible to modern manufacturing technologies. Using glasses derived from biomolecules, here, we created a variety of glass components by both 3D manufacturing and mold casting. Last, cyclic viscosity temperature curves were plotted to show the changes in η of the Ac-F system (fig. S27). The broad linear viscoelastic region of the Ac-F supercooled liquid provided accurate time windows for additive manufacturing, which may explain the flexible processability of these glasses.

### Biodegradation of biomolecular organic glasses in vitro and in vivo

**Fig. 5.**
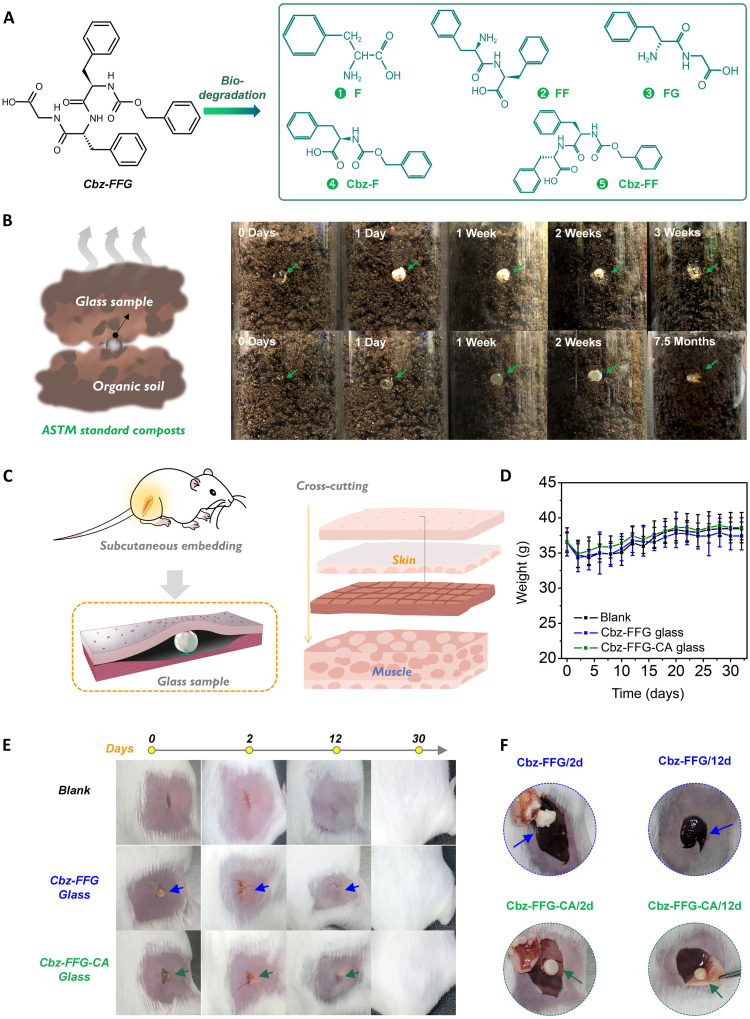
In vitro and in vivo degradation of glasses. (**A**) Enzymatic degradation products of Cbz-FFG glass identified by ESI-MS. Before the ESI-MS experiment, glass beads 0.3 cm in diameter were dipped in solutions (proteinase K solution, SGF_[sp]_, and SIF_[sp]_) and incubated at 37°C for 72 hours. (**B**) Degradation of glass beads with 0.5 cm diameter in composting soil consistent with ASTM standards. (**C**) Schematic diagram of glass subcutaneous implantation. Glass beads with a diameter of 0.35 mm were implanted between the skin and muscle layers. (**D**) Body weight versus time curve showing negligible weight loss after implantation of glass beads. (**E**) Photographs of glass degradation in vivo over time. (**F**) Anatomical data for mouse skin. The arrows indicate the position of glass.

To verify the biodegradability and biorecyclability of the developed glasses, we first investigated their degradation behaviors when exposed to proteinase K solution, simulated gastric fluid (SGF_[sp]_), and simulated intestinal fluid (SIF_[sp]_) (fig. S28). We observed that Ac-F and Cbz-FFG glass beads with 0.3 cm diameters were almost completely degraded after incubation in proteinase K solution for 1 week and 5 months, respectively. The Ac-F glass rapidly degraded upon immersion in the simulated enzymatic hydrolysates. After 4 hours of incubation, the size of the glass beads decreased notably, and after 2 days of incubation, the glass beads were no longer visible. By comparison, the degradation of Cbz-FFG glass required more time (approximately 5 months). We next sought to probe the degradation of the Cbz-FFG glass in more detail by using electrospray ionization mass spectrometry (ESI-MS) (Fig. 5A and figs. S29 and S30). The results suggested that the Cbz-FFG backbone degraded into several substructure units. Upon cleavage of amide links, Cbz-F and Cbz-FF were obtained, as verified by the emergence of peaks at 298.2 and 449.4, respectively. Concurrently, cleavage of the Cbz- modifier group yielded F, FF, and FG, as verified by the emergence of peaks at 167.9, 314.1, and 224.4, respectively. Cbz-FFG glass showed no obvious reducing or disappearing in water, due to the insoluble nature of the molecule (fig. S31), which can be inferred that the disappearance of Cbz-FFG glass in enzyme solutions is due to biodegradation but not direct dissolution.

Composting is one of the cost-optimal techniques for treating waste (*41*). To verify the compostable and biorecyclable of theses glasses, a composting device consistent with American Society of Testing Materials (ASTM) standards (*42*) was developed. Ac-F glass and Cbz-FFG glass both underwent degradation in soil composts (Fig. 5B). Ac-F glass notably decomposed in 3 weeks within the compost facilities. Cbz-FFG glass clearly disintegrated after being buried for 7.5 months. If properly planned and engineered landfills are built in appropriate locations, these glasses can be used as a sustainable energy source.

Encouraged by the promising results of in vitro degradation, we next explored the degradation of the developed glasses in vivo. Cbz-FFG glass bead and Cbz-FFG-CA glass bead encapsulating the immunosuppressive cyclic peptide cyclosporin A (CA) were implanted subcutaneously in mice (Fig. 5C). The survival status and body weight of the mice were monitored in real time. Throughout the experimental period, no mice exhibited any pain-related behavior that may have been induced by the glass implantation, and none of them experienced obvious weight loss (Fig. 5D). In addition, no symptoms of implant rejection, such as edema or exudation, occurred during the in vivo experiment. 

The degradation and inflammatory responses to Cbz-FFG glass and Cbz-FFG-CA glass were studied as follows. The skin surfaces and incisions were photographed on the 2nd, 12th, and 30th days after implantation to record the changes in the glasses (Fig. 5, E and F). The photographs showed that, at 2 days after implantation, the Cbz-FFG glass had obviously softened, while at 12 days after implantation, it was completely degraded and absorbed in vivo (Fig. 5F). The introduction of CA greatly prolonged the degradation time of the glass in vivo, which was attributed to the immunosuppressive effect of CA. Specifically, locally released CA is known to inhibit the proliferation and function of immune-related cells (e.g., T cells, B cells, and macrophages) to reduce the immune response (*43*). These glass implants were completely degraded 1 month after they were embedded. The skin of the mice recovered completely, and the hair around the embedding site grew effectively.

The tissues in contact with the glass implants, including the skin and muscle layers (Fig. 5C), were harvested and stained with hematoxylin and eosin (H&E) (fig. S32). Inflammatory cell infiltration (ICI) and cuticle thickening were observed in the mice in the blank group (skin incisions and wound closure) and the glass groups on the second day, which can be attributed to the initial tissue damage caused by surgery. H&E staining showed that the inward migration of muscle fiber cell nuclei and the distance between muscle cells increased, and these changes were accompanied by nonuniform cell sizes and shapes in the mice in the blank group and the Cbz-FFG group, suggesting that muscle fiber atrophy had occurred. However, these phenomena were not severe in the mice in the Cbz-FFG-CA glass group. ICI was greatly alleviated in mice on the 12th day, especially in mice in the Cbz-FFG-CA glass group, demonstrating the immunomodulatory effect and anti-inflammatory function of CA that was released upon glass degradation. On the 30th day, the structure of the muscle tissue appeared normal, the muscle fibers were arranged in an ordered manner, and no obvious ICI was observed. The above results revealed that the developed glasses display excellent biocompatibility and biosafety in vivo.

The side effects of bio-products or synthetic chemicals released into natural environments are a function of their composition, concentration, physicochemical speciation, and transformation ability (*44*). Amino acids and peptides are endogenous biomolecules and have long been recognized as completely eco-friendly and degradable. However, it is undeniable that the synthetic alkoxycarbonyl protective groups including Fmoc- or Cbz- are not native or endogenous to both microbes and the human body. Fortunately, these synthetic protective groups can be properly managed, transformed, and used, which can be mediated by properties of the environment. For example, the Fmoc-protected glycine can be used as corrosion inhibitor (*45*), and the Cbz-protected tyrosine is propagated as energy supplements (*46*). These biomolecular glasses, when coupled to rigorous on-demand degradation and sustainable utilization techniques, will offer opportunities to enhance their commercial value and application scope.

### Summary

We report the prototype of biodegradable and biorecyclable glasses composed of derivatives of naturally derived amino acids and peptides. This represents a critical step toward the development of eco-friendly glasses of biological origin beyond the currently used commercial glasses and plastic materials. These biomolecular glasses have excellent GFA and optical characteristics, and they are amenable to additive manufacturing. Our in vitro and in vivo experimental results reveal that these biomolecular glasses are biocompatible, compostable, biodegradable, and biorecyclable. Originating from naturally occurring amino acids or peptides, these biomolecular glasses with notable properties show advantage and promise for interfacing the biological and material worlds. This concept of biomolecular glass with biodegradability and biorecyclability represents an advanced demonstration of harmonious coexistence between humans and nature, and it is critical for tomorrow’s earth.

## MATERIALS AND METHODS

### Reagents

Amino acids were purchased or synthesized from Bachem, Macklin, Energy Chemical, J&K Scientific, GL Biochem (Shanghai) Ltd., or Aladdin Biochemical Technology Co. Ltd. Proteinase K from *Tritirachium album*, pepsin from porcine gastric mucosa, and pancreatin from porcine pancreas were purchased from Sigma-Aldrich. Hydrochloric acid (HCl), sodium hydroxide (NaOH), sodium chloride (NaCl), and monobasic potassium phosphate (KH_2_PO_4_) were products of Beijing Chemical Co. Ltd. The CQDs were purchased from Suzhou Xingshuo Nanotech Co. Ltd. The dyes Nile red, thioflavin T, rubrene, rhodamine B, and rhodamine 6G (R6G) were purchased from Sigma-Aldrich. CA was a product of Shanghai Yuanye Bio-Technology Co. Ltd. Composting soil was purchased from a local facility. Water was obtained from a double-stage Milli-Q Plus purification system (Millipore, America). All solutions were freshly prepared and filtered for immediate use.

### Glass transition and melting temperature analysis

The thermogravimetric curves and heat flow curves of all the samples were measured using a TGA/DSC 3+ series instrument (Mettler Toledo, Switzerland) and a DSC 1 instrument (Mettler Toledo, Switzerland). Before the tests, two empty platinum crucibles with loose lids were used for baseline adjustment. The amino acid or peptide powders were placed in one platinum crucible that was placed on the “*S*” side, and an empty crucible was placed on the “*R*” side as a control (Fig. 2A). By using the TGA/DSC 3+ series instrument, the samples were heated from room temperature to a relatively high temperature (e.g., ~500.15 K) to determine *T*_m_ and *T*_d_. For the DSC 1 instrument, the samples were heated and cooled following a preset procedure (Fig. 2B). For *Upscan* 1, the platinum crucible filled with powders was heated from room temperature (*T*_0_) to a temperature (*T*_1_) greater than *T*_m_ but less than *T*_d_. Quenching from *T*_1_ to a temperature (*T*_2_) lower than *T*_0_ was followed by an isothermal procedure at *T*_1_ for 15 min. For *Upscan* 2, the product was reheated from *T*_2_ to a temperature (*T*_3_) close to *T*_m_, and *T*_g_ was recorded. To ensure sample homogeneity, the platinum crucible was less than 50% full. The heating and cooling rates were 10 K min^−1^. Samples were protected by inert gases (e.g., argon) throughout the measurements.

### Glass formation

The glasses were formed by a heating and quenching procedure under an ultrapure inert gas atmosphere in a glove box (Mikrouna, China). The amino acid or peptide powder (50.0 mg) was placed in a clean glass container and heated. The samples were completely melted before being transferred to an experimental apparatus for quenching. For the heating process, the starting temperature was *T*_0_, and the ending temperature was *T*_1_. The quenching process followed an isothermal procedure for 15 min. For the quenching process, the final temperature was set at a temperature less than *T*_g._ The heating and cooling rates were 10 K min^−1^. The fully cured samples were used for subsequent characterization analysis.

### Viscosity-temperature test

Cyclic viscosity-temperature curves of Ac-F were measured using an MCR 302 rheometer (Anton Paar, Austria). The Ac-F powder (50.0 mg) was placed on the panel of the rheometer under an inert atmosphere, and the initial temperature was set to 473.15 K. The sample was heat-preserved for 15 min to melt the powder, and then the temperature was decreased to 273.15 to obtain a viscous liquid. The temperature was increased to 473.15 K and then lowered to 273.15 K, and this procedure was repeated for three cycles. The strain was 0.1%, and the heating and cooling rates were 10 K min^−1^.

### Calculations of ∆*C*_p_, enthalpy of fusion (∆*H*_m_), and *m*

The calculation of these thermodynamic and kinetic parameters was adapted from Wang *et al.* (*34*) with minor modifications. ∆*C*_p_ was calculated on the basis of the heat flow curve (*Upscan* 2) obtained by DSC by using Eq. 1.

$$\text{Δ}C_{p}\;(J\cdot K^{-1}\cdot g^{-1})=\frac{HF}{M\cdot V}$$ (1)

where HF is the heat flow, *M* is the amount of sample, and *V* is the heating or cooling rate. ∆*H*_m_ was calculated on the basis of the heat flow curve obtained by DSC-TGA by using Eq. 2.

$$\text{Δ}H_{m}\;(J\cdot g^{-1})=\frac{IA}{M}$$ (2)

where IA is the integrated area obtained by Origin 8.0 Software. *m* was calculated from Eq. 3.

$$m=\frac{56T_{g}\cdot \text{Δ}C_{p}}{\text{Δ}H_{m}}$$ (3)

### XRD and variable-temperature in situ XRD analysis

Room temperature XRD data were collected with a SmartLab (9 kW) diffractometer (Rigaku, Japan) equipped with a Cu filter and a Cu Kα1 radiation source (λ = 1.5406 Å). The collection conditions were as follows: a 2θ range of 10° to 45°, 10° min^−1^, and a step size of 0.01. The samples were placed in clean or silicon slices. For variable-temperature XRD measurements, a PANalytical Empyrean in situ diffractometer (PANalytical, The Netherlands) was used. The collection conditions were as follows: a 2θ range of 3° to 40°, 20° min^−1^, and a step size of 0.01. The samples were placed in a quartz cuvette with a 10.0 mm diameter and a 0.5 mm height.

### POM analysis

Cross-polarized microscopy images were acquired on a BX53 polarized microscope system (Olympus, Japan). The powder sample (1.5 mg) was gently placed on a slide with a diameter of 30.0 mm and a thickness of 1.0 mm. The powder sample was heated and cooled to form glass following the above method. Subsequently, the POM images were taken by microscopy.

### Raman spectroscopy

In situ Raman experiments of Ac-F single crystal cultivated following the literature method (*29*) were conducted on an RM1000 micro-Raman spectroscopy system (Renishaw, England). The spectra were collected in a backscattering geometry, and a grating of 1200 lines/mm was used. The collection conditions were as follows: laser wavelength of 532 nm as the excitation source and a range of 50 to 3500 cm^−1^. The samples were placed directly under the microscope without preprocessing, and data acquisitions were performed. Variable-temperature Raman spectra of Cbz-FFG were recorded using the LabRAM HR Evolution micro-Raman Spectrometer (Horiba, France) using 532-nm laser excitation in the backscattering geometry covering the wave number range of 400 to 3800 cm^−1^. The spectrometer resolution (per pixel) for a grating of 1800 lines/mm was ∼1.5 cm^−1^, and a 50× objective was used. In situ temperature-dependent Raman spectra were recorded from 298 to 458 K through three circles using a Linkam heating-cooling stage ensuring a temperature stability of ±0.1 K. The heating rate and cooling rate were set to be 10 K min^−1^.

### NMR spectroscopy

The solution ^1^H NMR spectra of digested samples (dissolved in dimethyl sulfoxide d6) were recorded on a 600-MHz Avance spectrometer equipped with a triple resonance cryogenic probe using a simple 1D pulse sequence (Bruker, Germany). ssNMR measurements were conducted on a 400-MHz Avance III system equipped with a 4-mm HXY triple resonance MAS probe (Bruker, Germany). The samples were ground by an agate mortar into a homogeneous powder, transferred to a zirconia NMR tube, and analyzed by the spectrometer.

### LCMS spectroscopy

We analyzed the composition changes before and after the glass formation using an Orbitrap mass spectrometry (Thermo Fisher Scientific, USA). The high-performance liquid chromatography–MS (HPLC-MS) conditions were listed as follows. For HPLC, the chromatographic separation was carried out in a Peptide BEH C18 column (2.1 × 150 mm, 1.7 μm) (Waters, USA). The used HPLC system was the Vanquish UHPLC (Thermo Fisher Scientific, USA). The mobile phase A and B were 0.1% formic acid–water and 0.1% formic acid–acetonitrile, respectively. The gradient elution procedure was performed as follows: 0 to 1 min, 5% B; 1 to 3 min, 5 to 40% B; 3 to 5 min, 40 to 100% B; 5 to 20 min, 100% B; 20 to 30 min, 5% B. The flow rate was 0.1 ml min^−1^. The injection volume was 5 μl. The column temperature was kept at 30°C. For MS, electron spray ionization positive mode was used. The spray voltage was 4.5 kV. The capillary temperature and vaporizer temperature were 320° and 300°C, respectively. The sheath gas and aux gas were 19.8 ml min^−1^ and 5 psi, respectively. The MS scan range was set from *m/z* 100 to 600. The samples including the powder and glass were dissolved in ethanol solution.

### Glass transmittance spectroscopy

The transmittance spectra of the Ac-F glass and the Cbz-FFG glass in the UV-visible region (200 to 800 nm) and NIR region (800 to 3000 nm) were obtained with a Cary 5000 UV-Vis-NIR spectrophotometer (Varian, USA). The glass was processed to a thickness of 1.0 mm and polished before analysis.

### Fluorescence spectroscopy

Solid fluorescence spectra were obtained using an RF-6000 spectrofluorometer (Shimadzu, Japan). The light source used in the spectrofluorometer was a 150-W Xe arc lamp (Ushio Inc., Japan). Scanning speed is 2000 nm min^−1^. Excitation bandwidth is 1.5 nm, and emission light bandwidth is 1.0 nm. The samples were placed in flat and clean quartz piece with quantitative groove. The ratio of amino acid derivatives to dye is 1.0 mol:0.05 mg.

### Computational simulations

The GROMACS package (version 5.1.4) was used to perform the AAMD simulations (*47*). The general AMBER force field (GAFF) was used to model the Ac-F molecule. To derive the force field parameters within the framework of the GAFF, the optimized geometry and molecular electrostatic potential of the Ac-F were obtained at the HF/6-31g (d) level of theory. The Antechamber package was then used to compute the partial charge according to the restrained electrostatic potential formalism (*48*). In this study, we performed AAMD simulations on 6 × 3 × 2 Ac-F lattices (*29*) with 144 Ac-F molecules. The system was first minimized using the conjugate-gradient algorithm with a tolerance on the maximum force of 200 kJ mol^−1^, and the temperature and volume of each system were equilibrated by running 400 ps of a constant-volume, constant-temperature (NVT) simulation followed by 400 ps of constant-temperature, constant-pressure (NPT) simulations. Last, the whole system was heated, maintained, and cooled to simulate the annealing process. The temperature change during the annealing process is shown in Fig. 3B. The leapfrog algorithm with a time step of 2 fs was used to integrate the equations of motion. The velocity rescale thermostat and the isotropic Parrinello-Rahman barostat were used with 0.4 and 2.0 ps as the thermostat and barostat relaxation times, respectively. The electrostatic forces were calculated by means of the particle-mesh Ewald approach with a cutoff of 1.0 nm. A 1.0-nm cutoff was also used for the van der Waals forces. The LINCS algorithm was applied at each step to preserve the bond lengths.

### 3D printing and model pouring experiment

Powders of Ac-F and CQDs or dyes were mixed evenly before printing or casting in molds. The mass ratio of CQDs or dyes to Ac-F is 1:10^5^. We printed 3D structures using a 3D Discovery bioprinter (RegenHU, Switzerland) in a laminar-flow hood. The mixture was used to fill the charging barrel. The barrel temperature was raised to 500.15 K, and the flow was regulated by pressure (3.5 bar). The print head (HM 300H) operated in the *y*, *z* plane, and the collector operated along the *x* axis. The sample mixture was placed in a special glass dish, which was heated in a high temperature furnace with an operation temperature of 500.15 K to obtain a supercooled liquid. The liquid was slowly poured into the molds. Next, the molds were transferred to a low-temperature device with an operation temperature of 273.15 K and demolded after complete solidification.

### Characterization of glass degradation in vitro

Degradation was conducted in an enzyme solution (0.1 mg ml^−1^ proteinase K solution at pH 7.50) at a specified temperature (37° to 38°C). Further degradation experiments were conducted using solutions of SGF_[sp]_ and SIF_[sp]_, which were prepared according to the United States Pharmacopeia. For SGF_[sp]_ (pH ≈1.2), 1.0 g of NaCl and 1.6 g of pepsin were dissolved in 3.5 ml of HCl and sufficient water to make a 500-ml test solution. For SGF_[sp]_, 6.8 g of KH_2_PO_4_ was dissolved in 250 ml of water and mixed, and 77.0 ml of 0.2 N NaOH and 500 ml of water were added. Next, 10.0 g of pancreatin was added, the solution was mixed, and the pH was adjusted to 6.8. Then, the solution was diluted with water to 1000 ml. Glass beads with a diameter of 0.3 cm were dipped in the above solutions and incubated at 37°C. Photographs were taken to record the morphological changes. ESI-MS spectrometry (APEX II, Bruker, Germany) was used to identify the degradation products of the Cbz-FFG glasses. The Cbz-FFG glass beads were incubated with 2.5 ml of proteinase K solution, SGF_[sp]_, and SIF_[sp]_ at 37°C for 2 weeks. The solution was analyzed by ESI-MS after being filtered through a 0.45-μm filter membrane.

### Degradation in composts

Soil was purchased from a local composting company. The soil was baked overnight at 120°C, and then water was added to the dry soil to achieve a total moisture content of 50%, consistent with ASTM standards. Glass beads with a diameter of 0.5 cm were embedded in the soil with a depth of 2.5 cm. To facilitate observations, the compost soil was placed in transparent glass containers.

### Characterization of glass degradation in vivo

All in vivo experiments were handled in accordance with the protocols approved by the Institutional Animal Care and Use Committee (IACUC) in compliance with Chinese law for treatment of experimental animals. The Ethics Committee for Animal Experimentation of Institute of Process Engineering, CAS, approved the study. Four- to 6-week-old female Kunming mice weighing approximately 30 g each were purchased from Vital River Laboratory Animal Technology Co. Ltd. (Beijing, China). The mice were kept in a specific pathogen–free facility with plenty of food and water. Before the experiments, each mouse was injected with anesthesia (pentobarbital, 50.0 mg kg^−1^). Immediately afterward, the dorsal areas of the mice were shaved and disinfected with 75% ethanol, and incisions (approximately 0.5 to 0.6 cm) were made. Cbz-FFG glass beads with a diameter of 0.35 mm were gently placed into the connective tissues. The mass percentage of CA in the Cbz-FFG-CA glass was 10%. CA powder was added to the viscous liquid of Cbz-FFG, and after stirring and dissolving evenly, the mixture was cooled to form Cbz-FFG-CA glass. The skin surface at the incision sites was sutured using suture string (5-0 type with a wire diameter of ~0.1 mm). At 2, 12, and 30 days after embedding the glass beads, the mice were anesthetized with an isoflurane and gas-air mixture. Tissues surrounding the embedding sites, including the skin and muscle (Fig. 5C), were excised and harvested. The tissues were washed with phosphate-buffered saline and fixed in a 4% paraformaldehyde solution. Then, the tissues were embedded in paraffin blocks, sectioned into 5.0-μm slices, and mounted on glass slides. Standard H&E staining was performed, and the samples were analyzed by professional technicians according to International Organization for Standardization (ISO) guidelines for evaluating the local effects of materials. The body weights of the mice were recorded every day, and photographs were taken.
