# Supplementary material for: Biomolecular glass with amino acid and peptide nanoarchitectonics
Source: Sci Adv. 2023 Mar 17;9(11):eadd8105. doi: 10.1126/sciadv.add8105 (PMC10022897; doi:10.1126/sciadv.add8105)

Science Advances
AAAS

Supplementary Materials for

Biomolecular glass with amino acid and peptide nanoarchitectonics

Ruirui Xing *et al.*

Corresponding author: Xuehai Yan, yanxh@ipe.ac.cn

Sci. Adv. **9**, eadd8105 (2023)
DOI: 10.1126/sciadv.add8105

This PDF file includes:

Tables S1 to S3
Figs. S1 to S32

Tab. S1.

Kinetic and thermodynamic parameters related to the glass transitions of amino acid– and peptide–based glasses. The parameters include the glass transition temperature (T_g), melting temperature (T_m), decomposition temperature (T_d), reduced glass transition temperature T_{rg} (T_g/T_m), glass transition heat capacity increase (ΔC_p), enthalpy of fusion (ΔH_m), and fragility m–index (*m*). These characteristic parameters were determined with heating and cooling rates of ±10 K min^{-1}.

Amino Acid or Peptide Derivatives	T_g (K)	T_m (K)	T_d (K)	T_{rg}	ΔC_p ($J \cdot K^{-1} \cdot g^{-1}$)	ΔH_m ($J \cdot g^{-1}$)	*m*
Ac–Q	307.90	477.80	591.73	0.64	1.10	443.07	43
Ac–G	293.40	484.80	515.77	0.61	0.38	409.24	15
Ac–H	365.00	412.90	532.85	0.88	0.91	428.20	43
Ac–F	312.46	442.30	534.50	0.71	0.70	258.87	48
Ac–Y	328.80	427.20	541.25	0.77	2.22	522.07	78
Fmoc–G	313.50	450.80	541.35	0.70	0.69	242.98	50
Fmoc–F	309.00	460.10	531.70	0.67	0.78	231.78	58
Fmoc–V	304.70	423.00	529.65	0.72	0.71	200.25	61
Cbz–Y	313.20	370.95	548.75	0.84	0.72	209.76	60
Cbz–F	303.40	360.10	537.28	0.84	0.13	171.71	13
Cbz–FF	316.65	427.65	544.50	0.74	0.61	533.20	20
Cbz–YY	349.35	406.85	541.40	0.86	0.57	567.05	20
Cbz–YL	330.65	424.75	543.73	0.78	0.63	280.82	42
Cbz–FFG	333.05	453.28	519.80	0.73	0.67	199.84	63

Tab. S2.

Photographs of amino acid–based glasses. A series of amino acids modified using Ac–, Fmoc– and Cbz– groups show the ability to form a glass through the classic heating–quenching procedure.

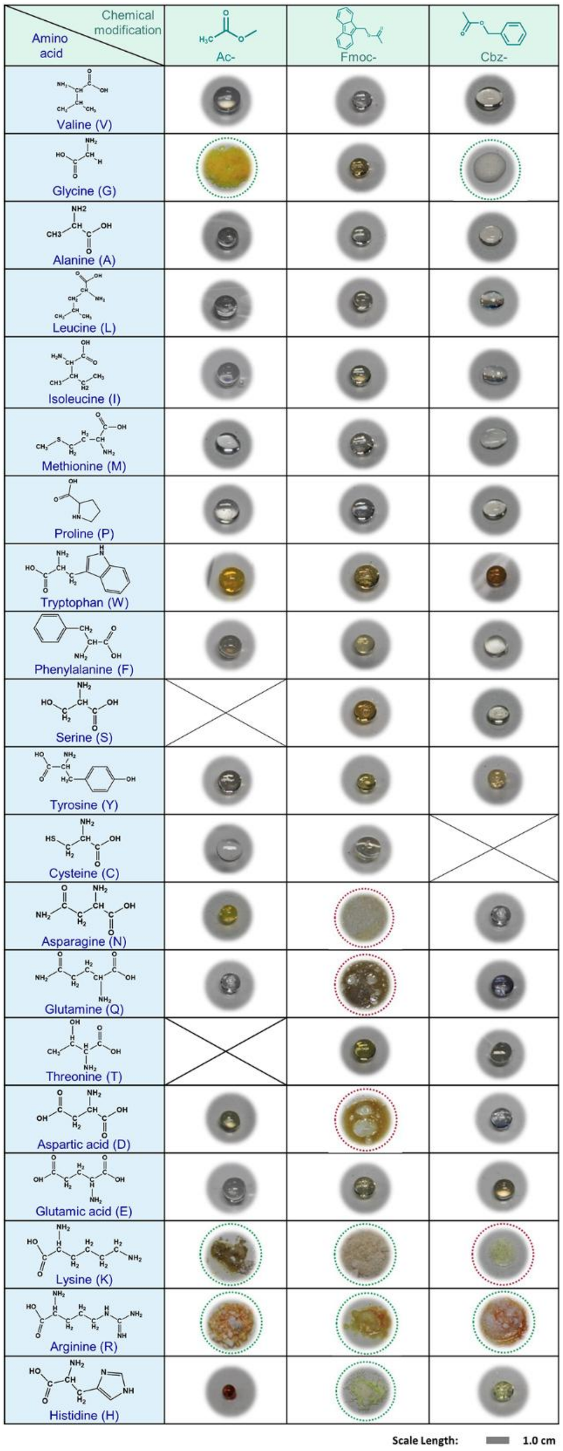

*The green circles indicate amino acid derivatives that have not yet formed a glass through the melting and quenching processes. The red circles indicate amino acid derivatives that formed glass analogues, but their processability must be improved. Because of the limitations of synthetic technology, Ac–S, Ac–T and Cbz–C were not used in this manuscript.

*It should be noted that these raw materials were exchanged in the oxygen–free glove box for 3 months, but the oxygen adsorbed onto the powders could not be completely eliminated.

Tab. S3.

Single crystal data of Ac–F. Crystal data and structure refinement of Ac–F single crystal.

**Crystal data and structure refinement of Ac–F**	
Identification code	Ac–F
Empirical formula	$C_{11}H_{13}NO_3$
Formula weight	207.22
Temperature/K	169.99 (10)
Crystal system	orthorhombic
Space group	$P2_12_12_1$
a/Å	5.64524 (6)
b/Å	11.14117 (12)
c/Å	16.96905 (17)
α/°	90
β/°	90
γ/°	90
Volume/Å^3	1067.261 (19)
Z	4
ρ_{calc}g/cm^3	1.290
μ/mm^{-1}	0.780
F (000)	440.0
Crystal size/mm^3	0.18 × 0.11 × 0.03
Radiation	Cu Kα (λ = 1.54184)
2θ range for data collection/°	9.496 to 151.054
Index ranges	$-6 \leq h \leq 7$, $-13 \leq k \leq 13$, $-21 \leq l \leq 21$
Reflections collected	13465
Independent reflections	2191 [R_{int} = 0.0263, R_{sigma} = 0.0153]
Data/restraints/parameters	2191/0/139
Goodness–of–fit on F^2	1.116
Final R indexes [I>=2σ (I)]	R_1 = 0.0271, wR_2 = 0.0728
Final R indexes [all data]	R_1 = 0.0276, wR_2 = 0.0732
Largest diff. peak/hole / e Å^{-3}	0.15/–0.16
Flack parameter	0.04 (6)

Fig. S1.

Thermogravimetric analysis and enthalpic responses of amino acids without protecting groups using a DSC–TGA instrument. The measurement indicates different degrees of decomposition of (A) Glutarnine, (B) Histidine, (C) Phenylalanine, (D) Tyrosine, (E) Valine and (F) Leucine at T_m. The cooling or heating rate was ±10 K min^{-1}. The vertical lines denote the starting and ending points of weight loss around T_m, respectively.

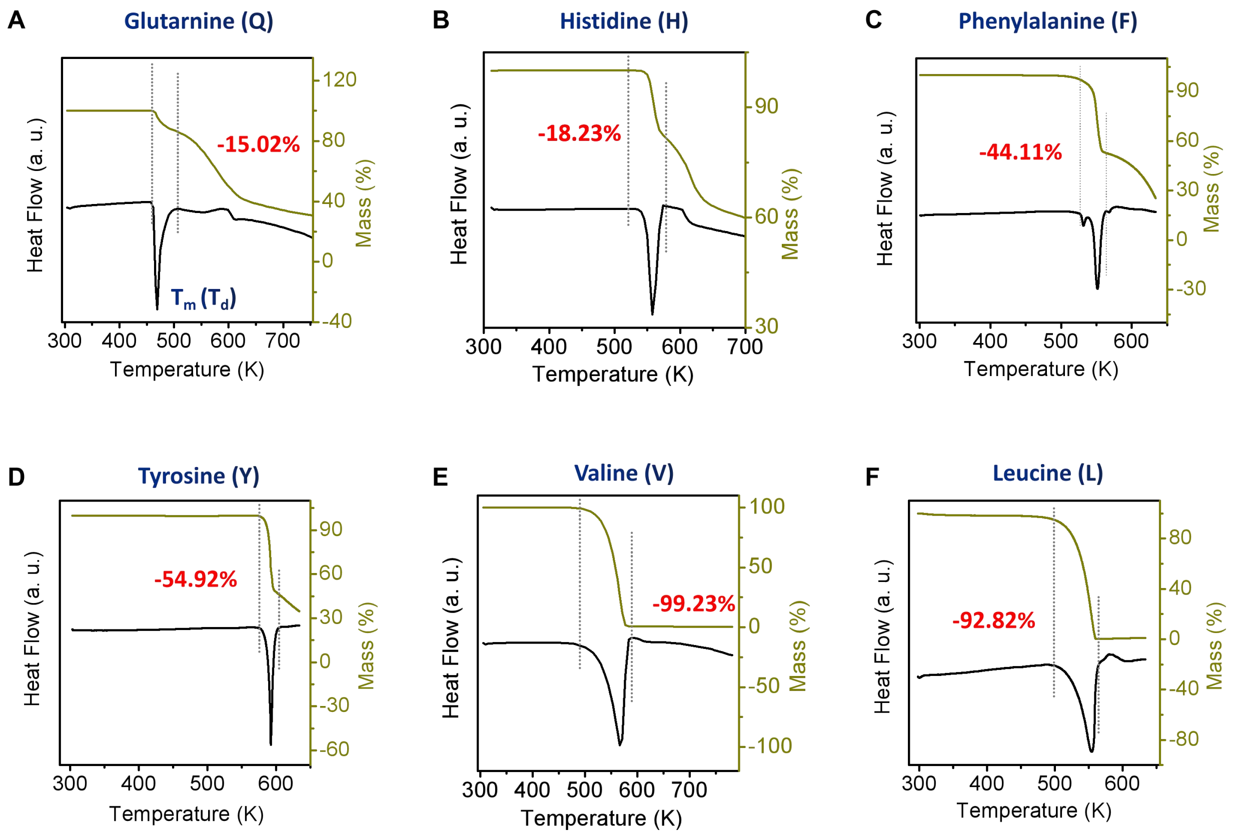

Fig. S2.

A peptide glass. Molecular structure of Cbz–FFG and photograph of the corresponding glass.

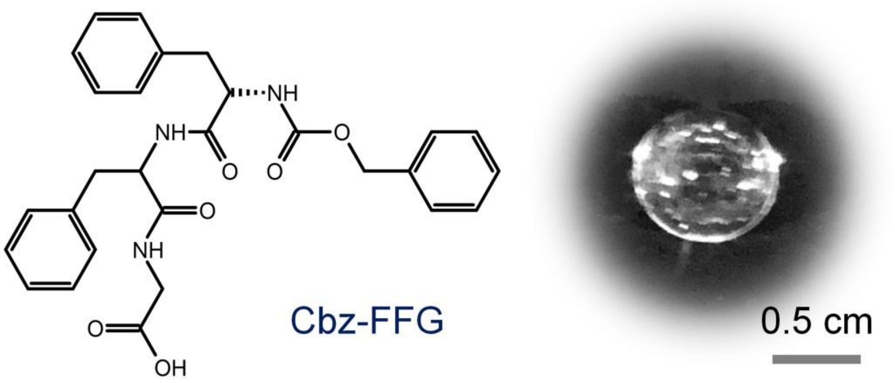

Fig. S3.

XRD patterns of typical amino acid or peptide powders (blank line) and glasses (red line), showing the obvious crystalline nature of the raw material powders and the amorphous nature of the as–prepared glasses.

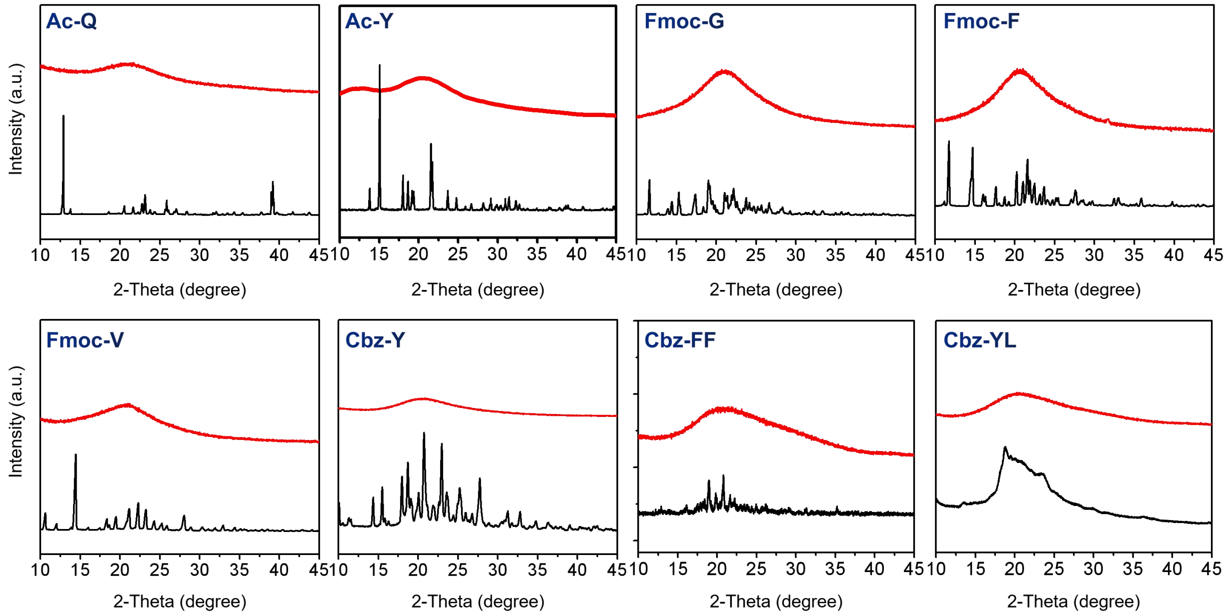

Fig. S4.

The DSC–TGA results of Fmoc–W and Cbz–W. The red arrow represents the occurrence of oxidation and volatilization.

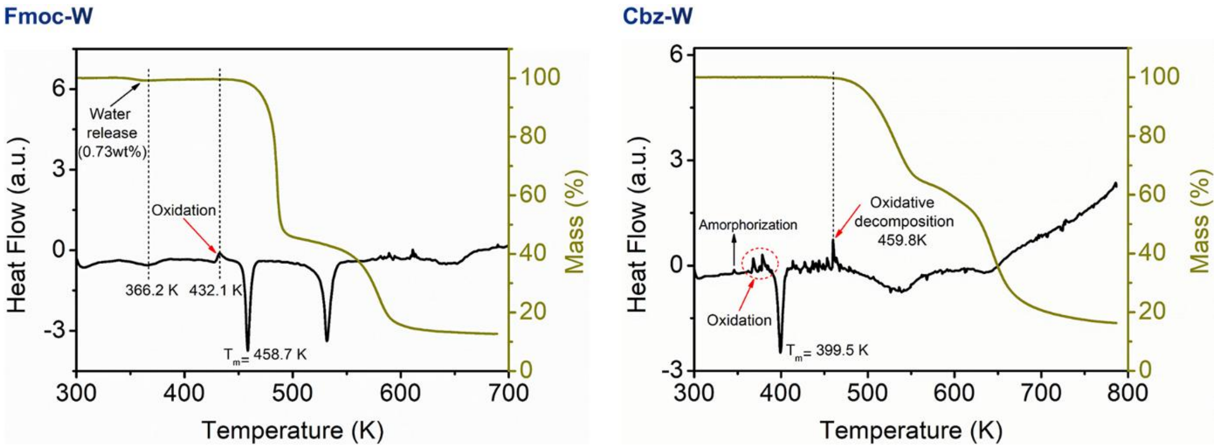

Fig. S5.

LCMS of Fmoc–W before and after the glass formation (data from positive–ion mode shown). Upper: the chromatogram of Fmoc–W showing the m/z of 427.1650 as a function of retention time; Middle: the molecular structure and m/z of Fmoc–W; Bottom: the chromatograms of Fmoc–W powder and Fmoc–W glass, as well as the % Area data of quantitative analysis, showing a 5.23% integral area loss of Fmoc–W.

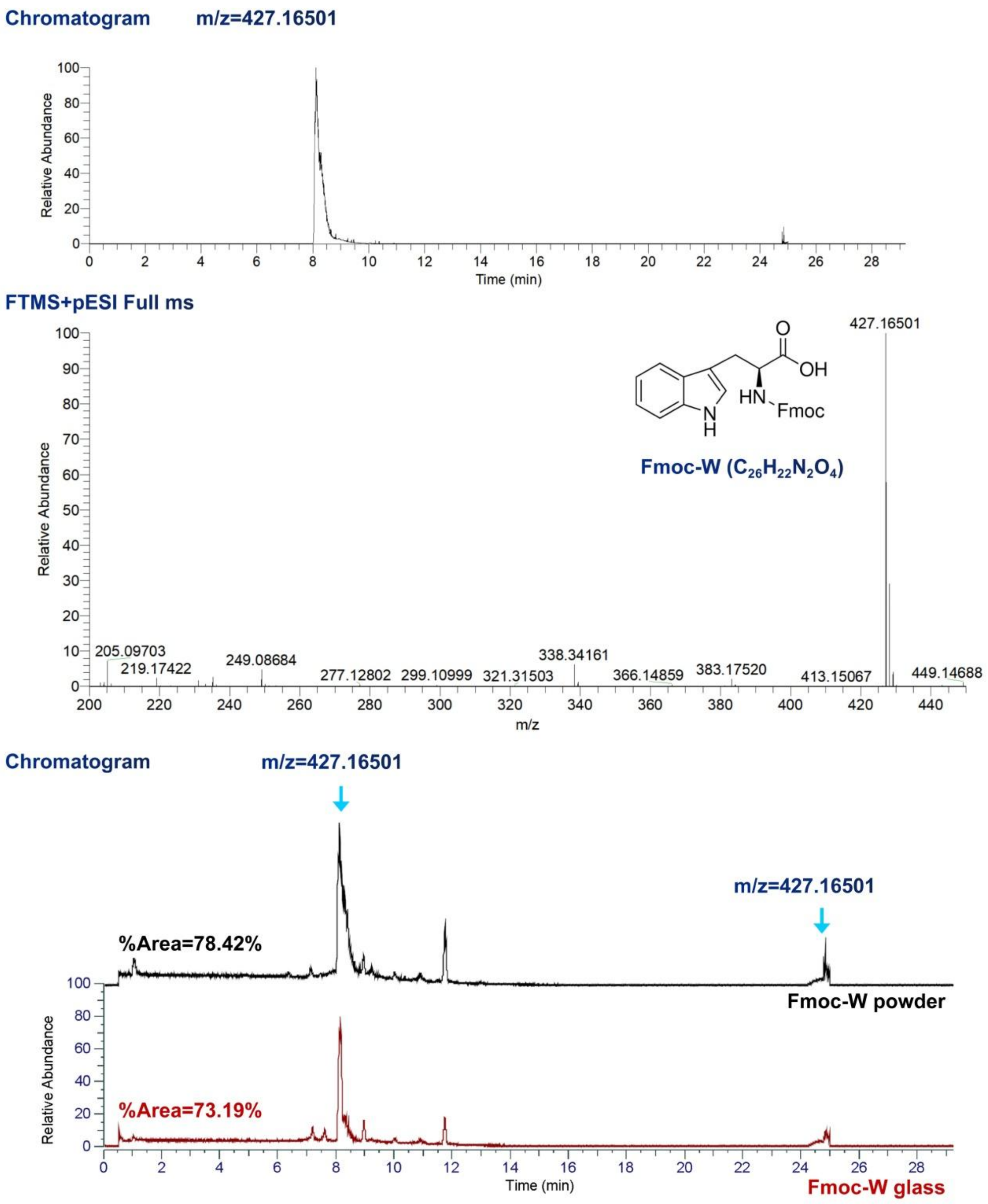

Fig. S6.

LCMS of Cbz–W before and after the glass formation (data from positive–ion mode shown). Upper: the chromatogram of Cbz–W showing the m/z of 339.1339 as a function of retention time; Middle: the molecular structure and m/z of Cbz–W; Bottom: the chromatograms of Cbz–W powder and Cbz–W glass, as well as the % Area data of quantitative analysis, showing a 2.96% integral area loss of Cbz–W.

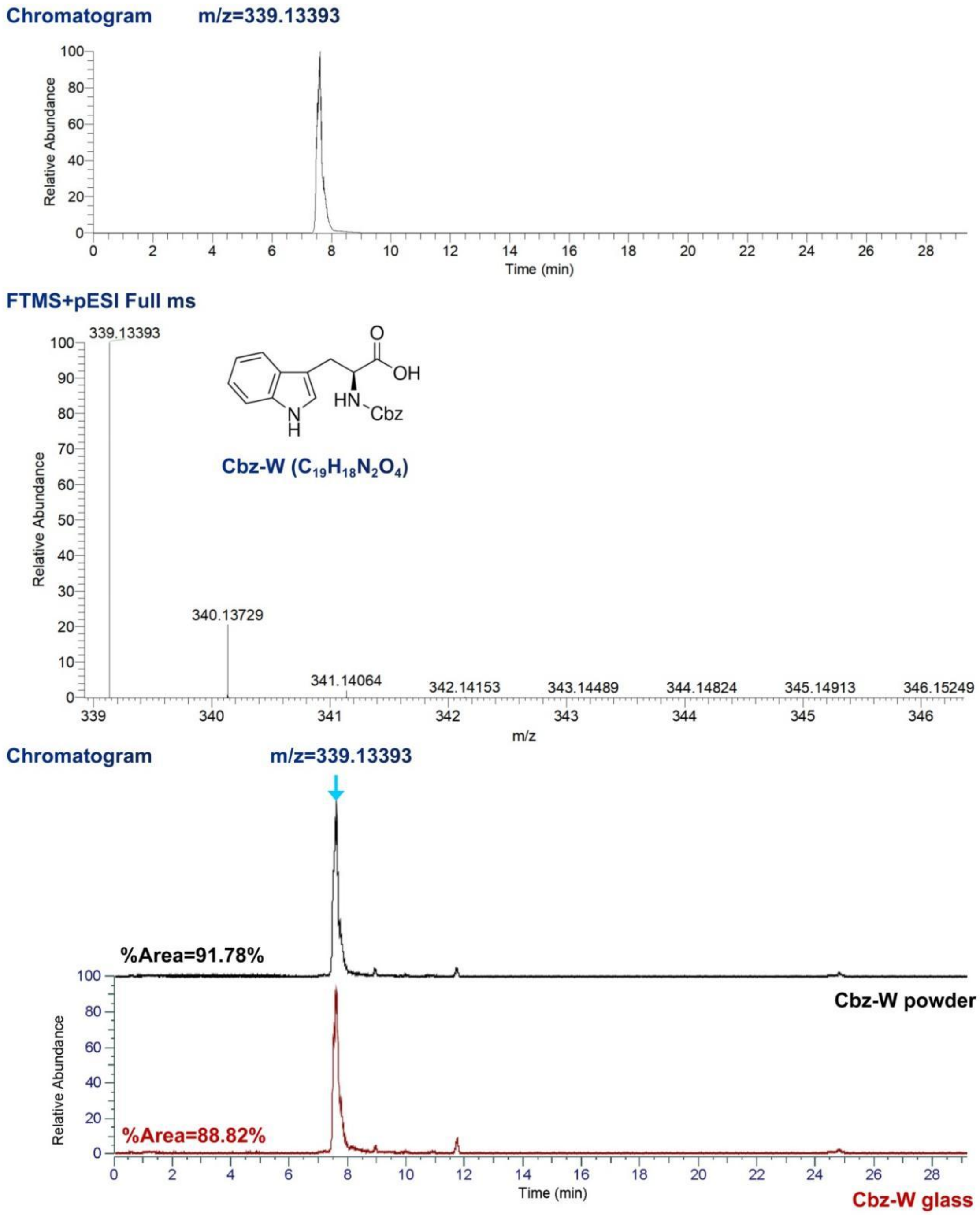

Fig. S7.

The DSC–TGA result of Ac–N. The result shows no evidence of qualitative decomposition or volatilization at T_m during the glass formation.

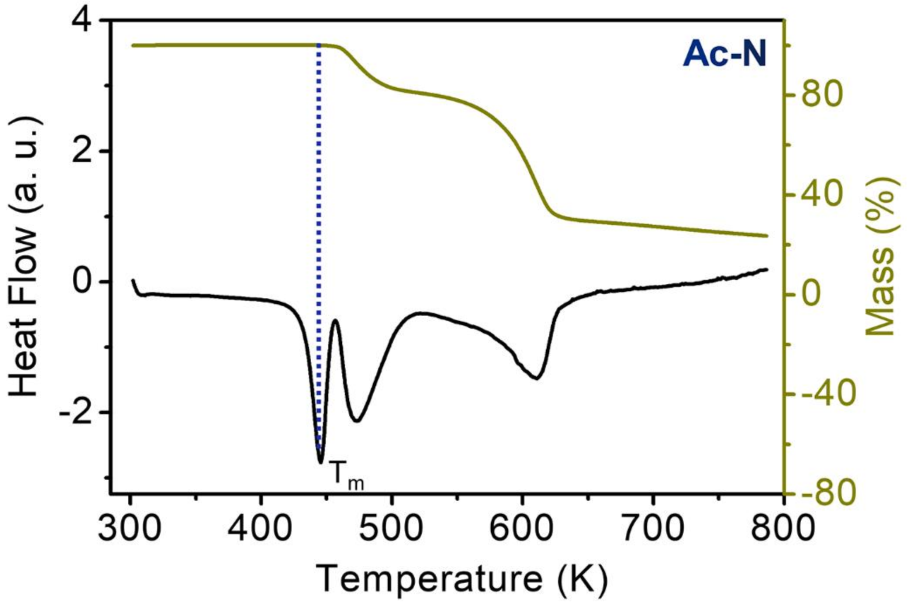

Fig. S8.

LCMS results of Ac–N (data from positive–ion mode shown). Upper: the chromatogram of Ac–N showing the m/z of 175.0713 as a function of retention time; Middle: the molecular structure and m/z of Ac–N; Bottom: the chromatograms of Ac–N powder and Ac–N glass, as well as the % Area data of quantitative analysis, showing no integral area loss of Ac–N.

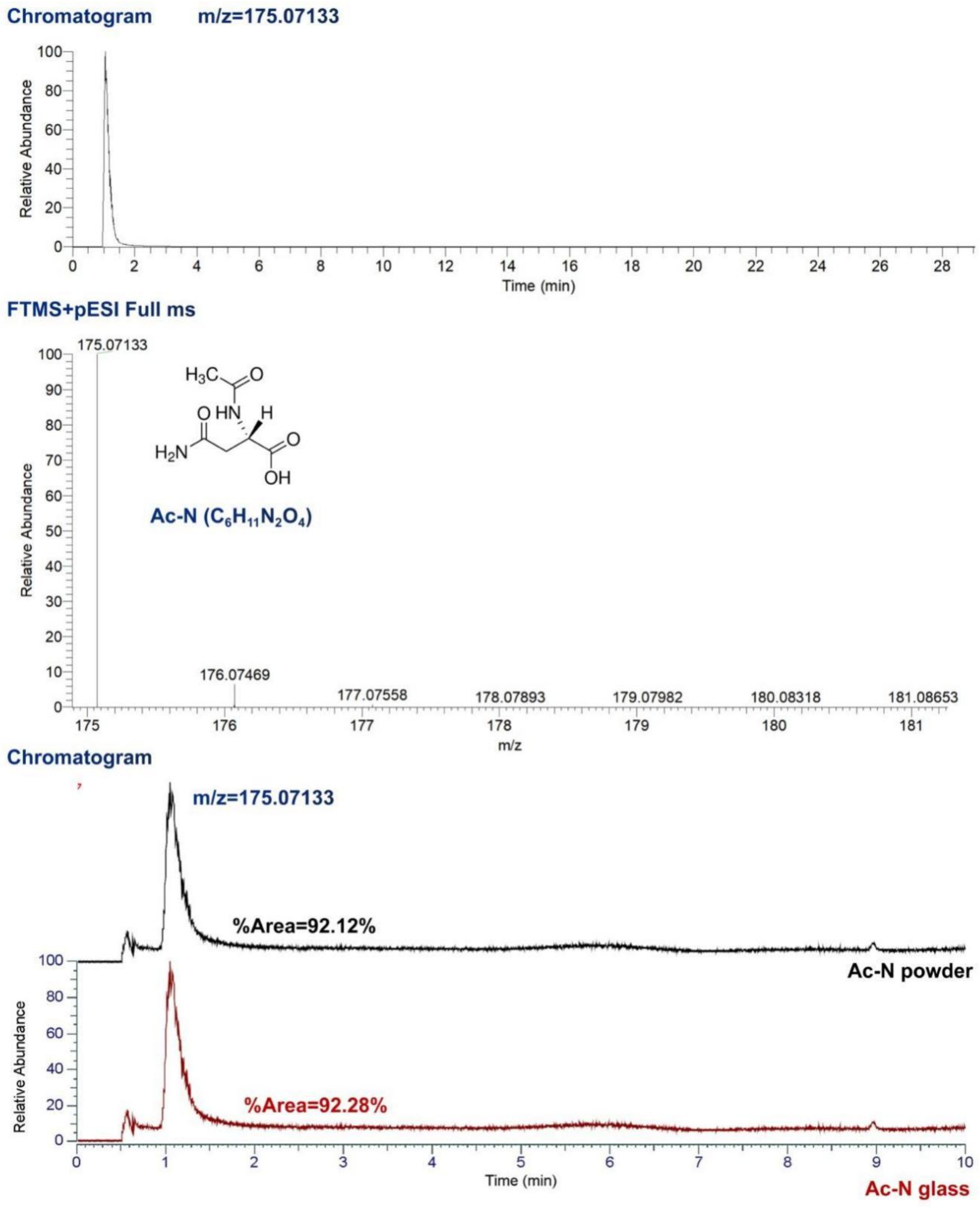

Fig. S9.

The DSC–TGA results of Cbz–R, showing the obvious mass loss near T_m that is quite close to T_d.

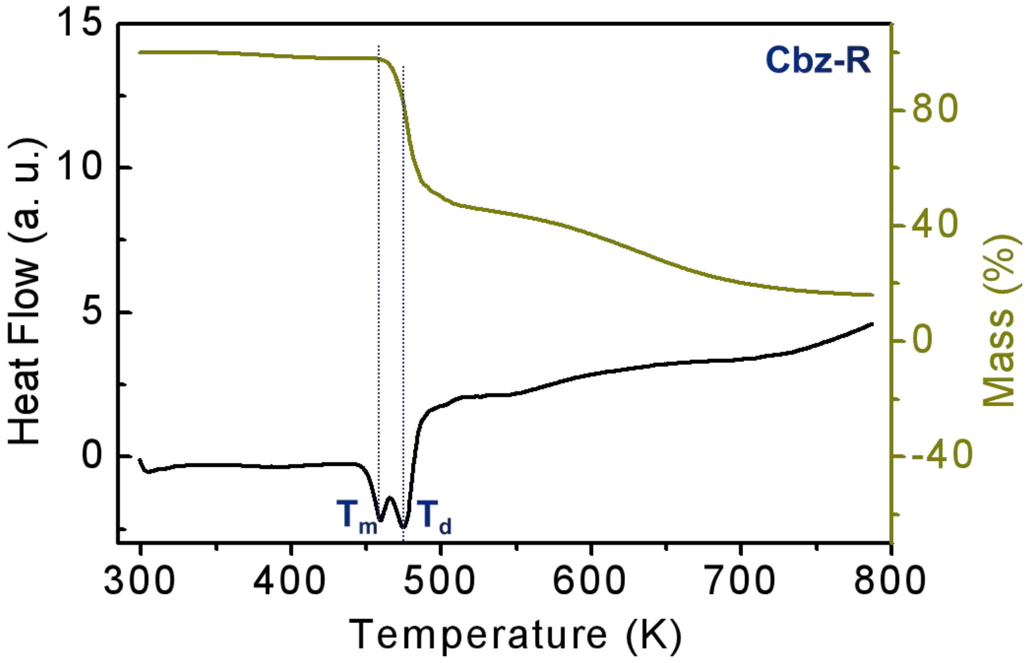

Fig. S10.

LCMS results of Cbz–R before and after heating (data from positive–ion mode shown). The m/z and probable molecular structures of decomposition and oxidation products are assigned in Cbz–R sample. Upper: the chromatogram of Cbz–R showing the m/z of 309.1557 as a function of retention time; Middle: the molecular structure and m/z of Cbz–R; Bottom: the chromatograms of Cbz–R powder and Cbz–R sample after heat treatment, as well as the % Area data of quantitative analysis, showing remarkable integral area loss of Cbz–R.

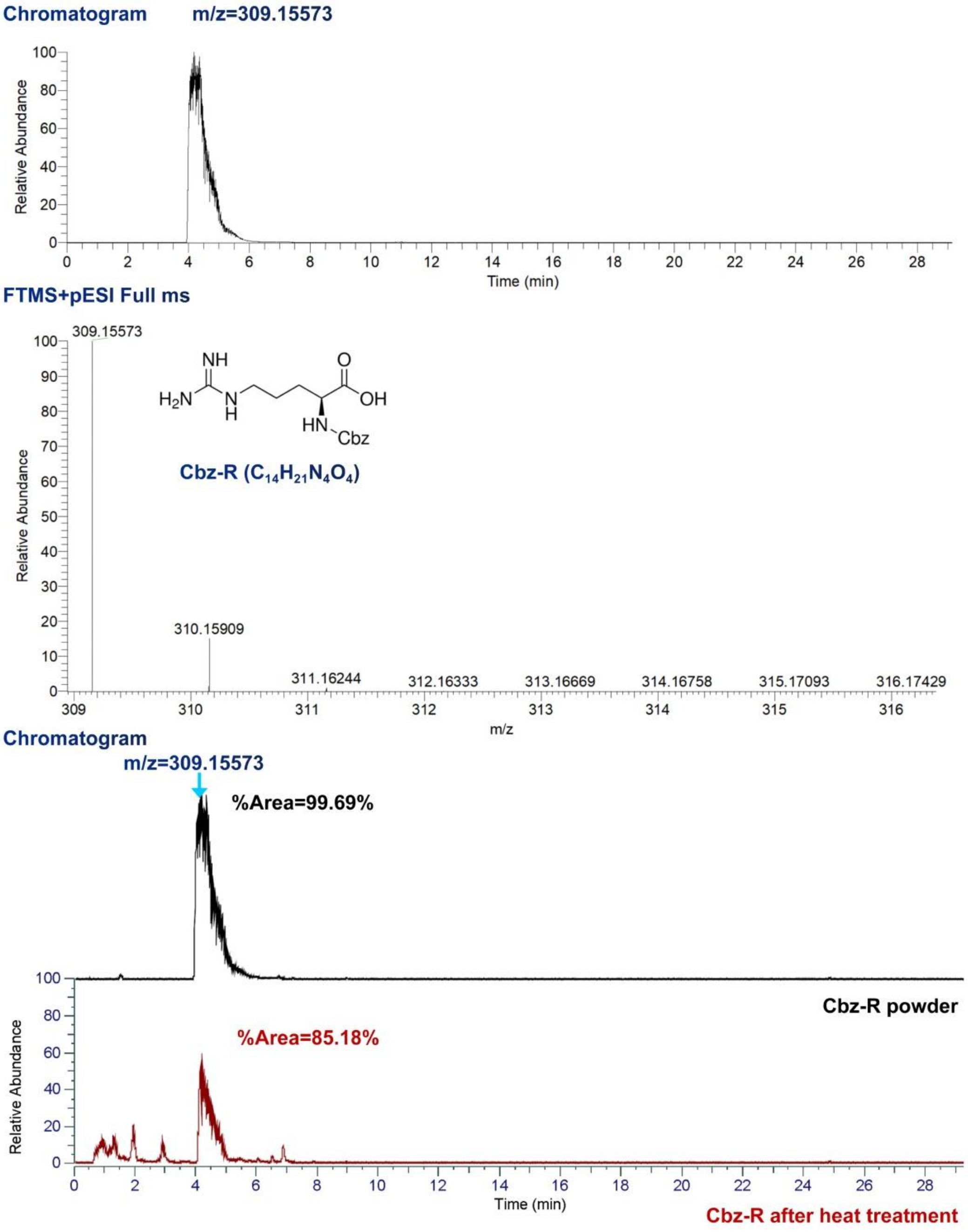

Fig. S11.

LCMS results of Cbz–R after heat treatment. The m/z and probable chemical formula of decomposition and oxidation products are assigned in Cbz–R sample. RT values denote the retention time corresponding to the above products.

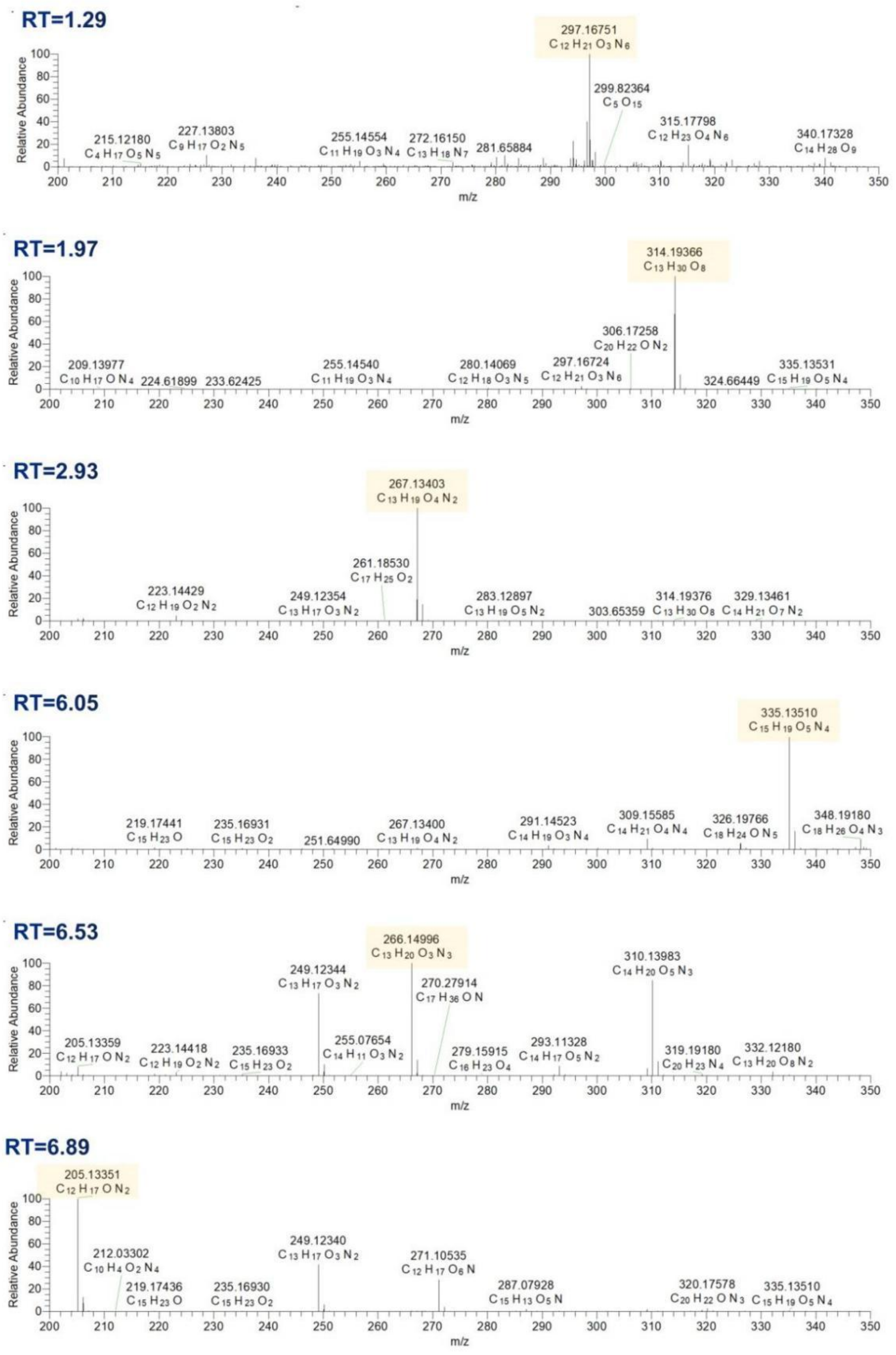

Fig. S12.

***In situ* XRD patterns during the Ac-F glass formation.** The transformation of Ac–F from the crystalline state to the glassy state during heating–quenching across the entire temperature ranges was recorded.

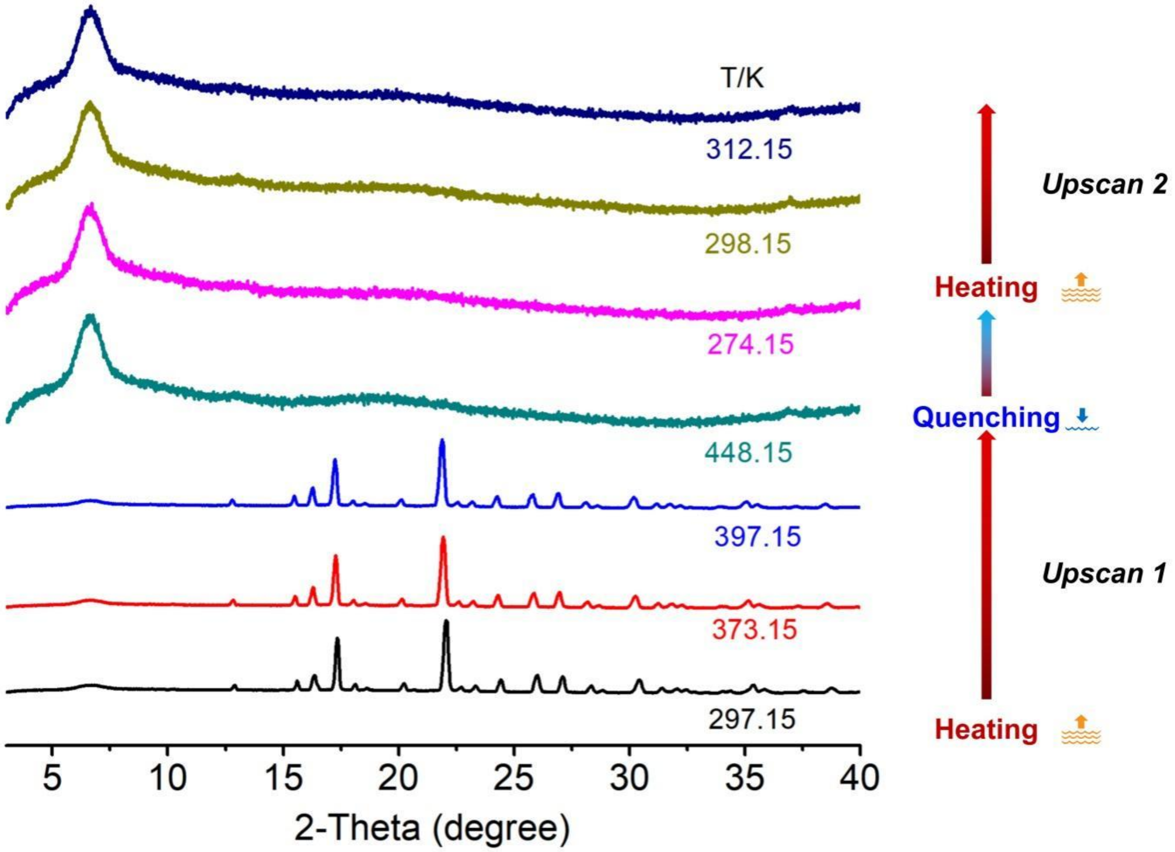

Fig. S13.

Solid–state NMR (ssNMR) spectra of Ac–F samples, indicating no change in the Ac–F chemical structure during the formation of a glass.

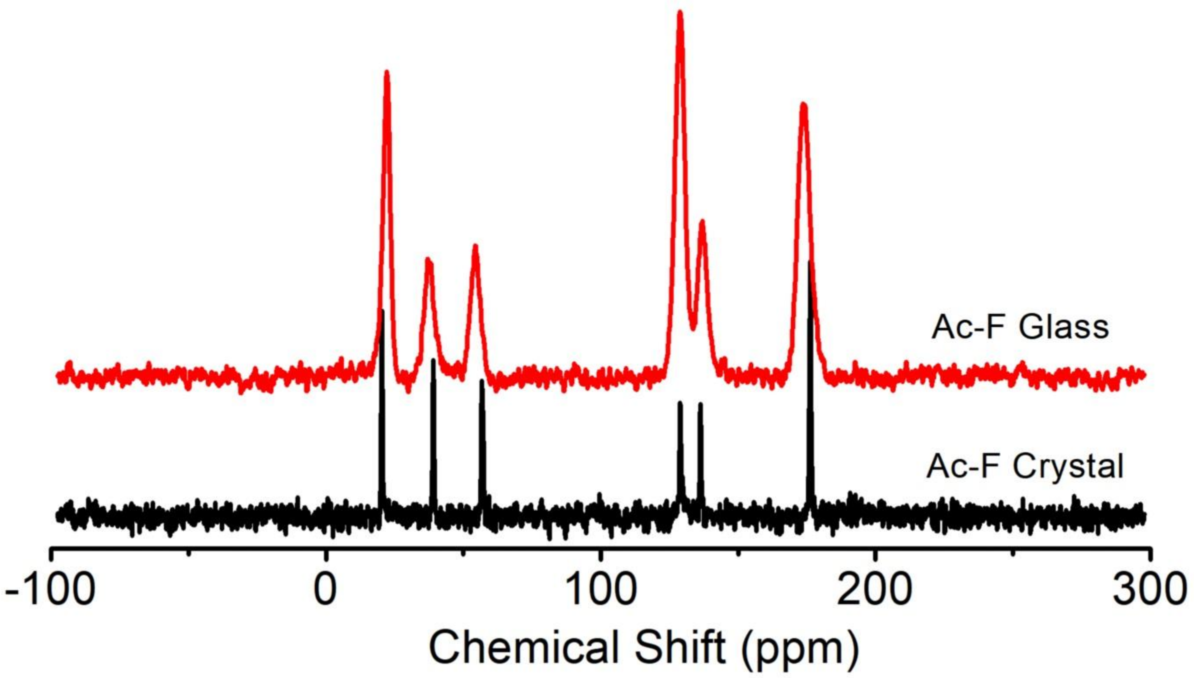

Fig. S14.

***In situ* Raman spectra of Cbz–FFG as a function of temperature.** Cbz–FFG powder was heated, melted and then quenched to form a glass. The cooling or heating rate was ±10 K min^{-1}.

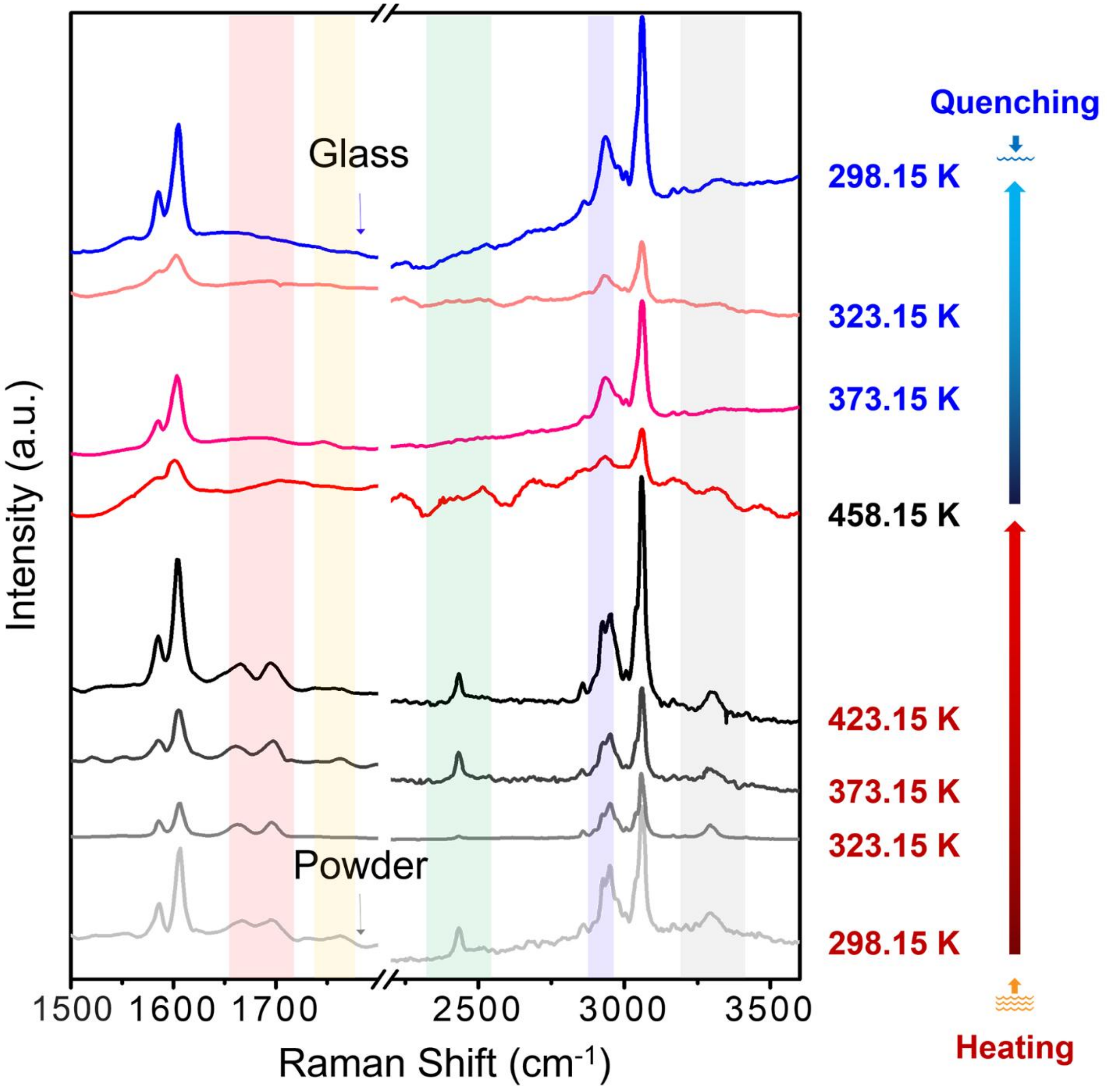

Fig. S15.

***In situ* Raman spectra of Cbz–FFG as a function of temperature in successive "heating–cooling" cycles.** The cooling or heating rate was ±10 K min^{-1}. The result indicated that the molecular arrangements at each stage of glass formation (including heating, melting, and cooling) were not changed after successive heating-cooling cycles.

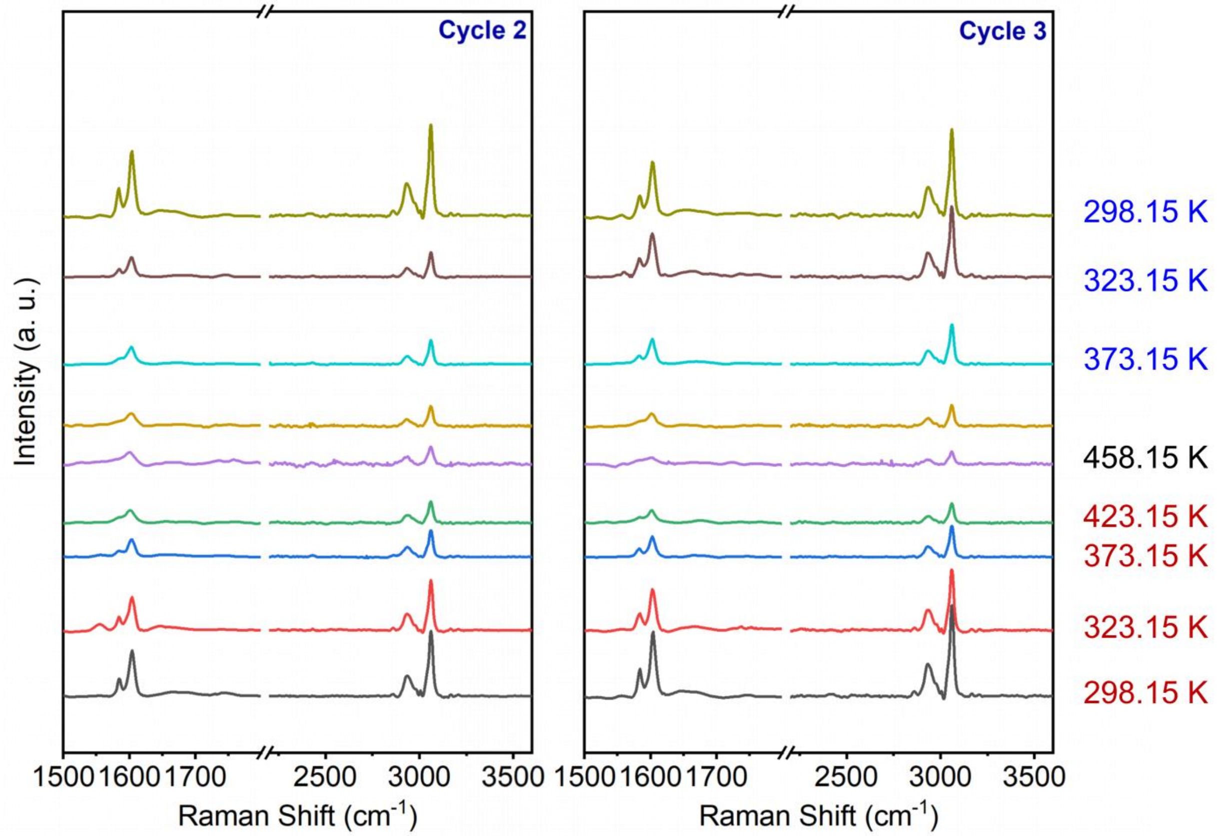

Fig. S16.

LCMS results of Cbz–FFG powder (data from positive–ion mode shown). Upper: The chromatogram of Cbz–FFG powder as a function of retention time and the % Area data of quantitative analysis of Cbz–FFG (the m/z is 504.2130); Bottom: the molecular structure and m/z of Cbz–FFG (blue arrow).

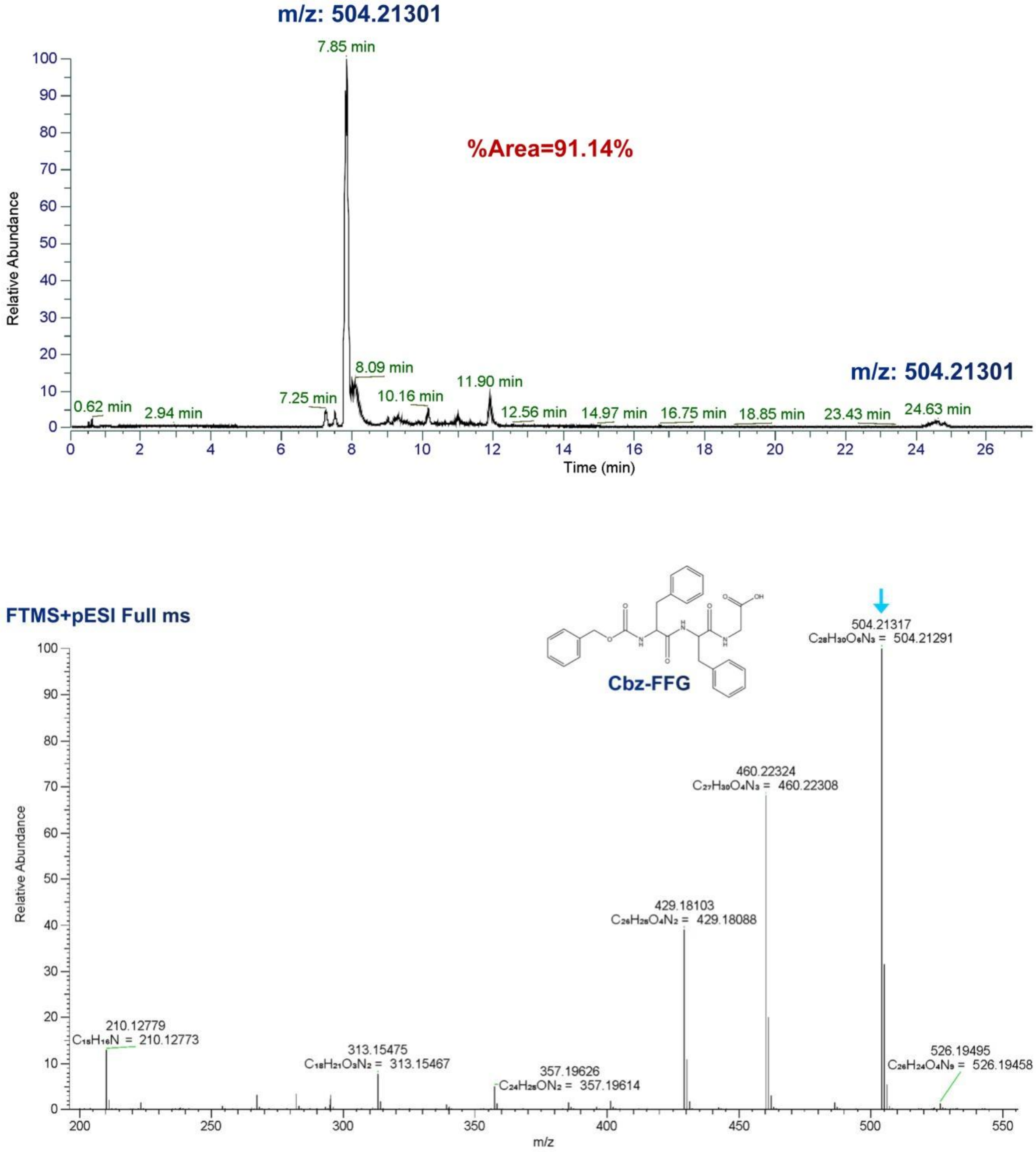

Fig. S17.

LCMS results of Cbz–FFG glasses (data from positive–ion mode shown). The chromatogram of Cbz–FFG glasses as a function of retention time and the % Area data of quantitative analysis of Cbz–FFG glasses, which shared the same m/z of Cbz–FFG powder. It was noted that the Cbz–FFG glasses have undergone one to three cycles of reproducing.

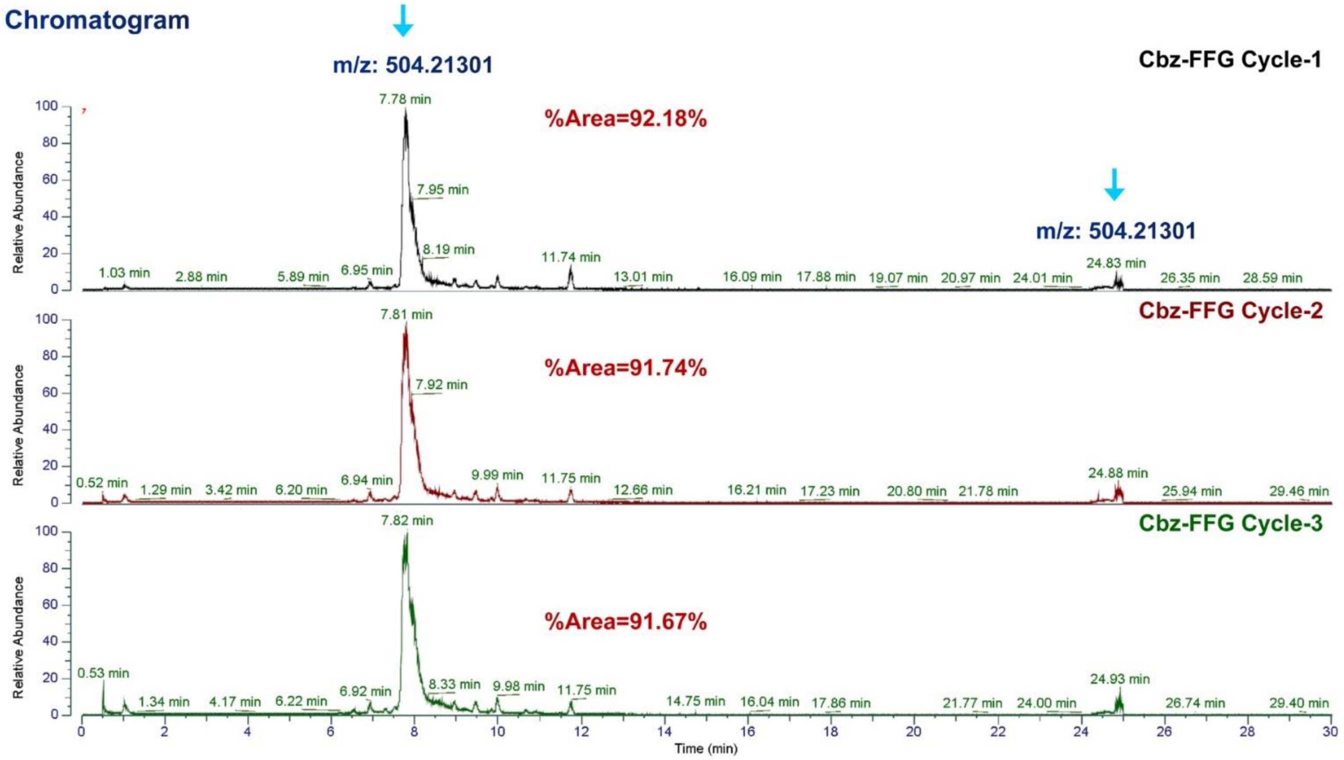

Fig. S18.

Hydrogen bond types in the (A) single crystal and (B) glass of Ac–F, indicating that multiple types of hydrogen bonds, especially O2–H···O3 and N–H···O1, appeared and became more abundant after the formation of glass.

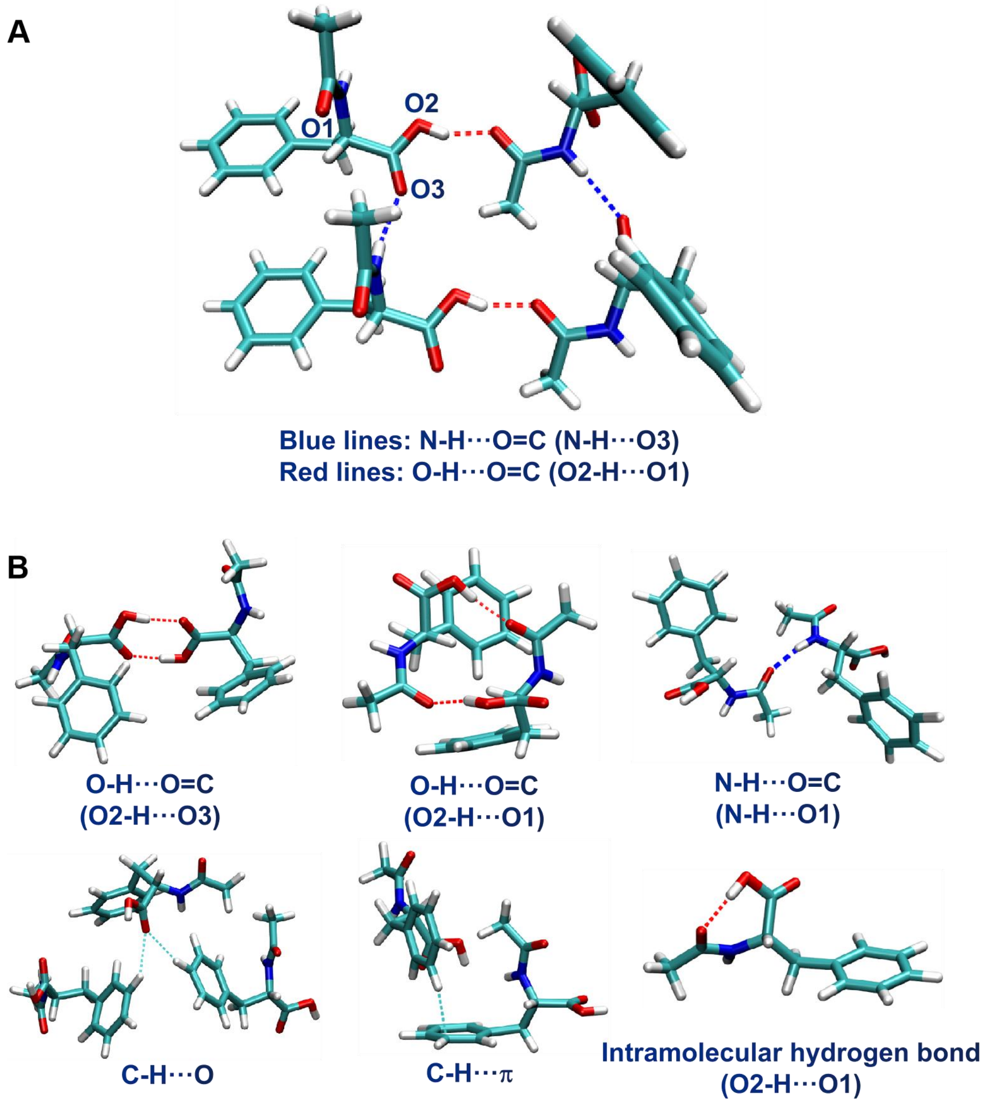

Fig. S19.

The XRD patterns of Ac–F crystal formed at a cooling rate of 0.5 K min^{-1}, showing the Bragg diffraction patterns different from Ac–F single crystal obtained from Table S3.

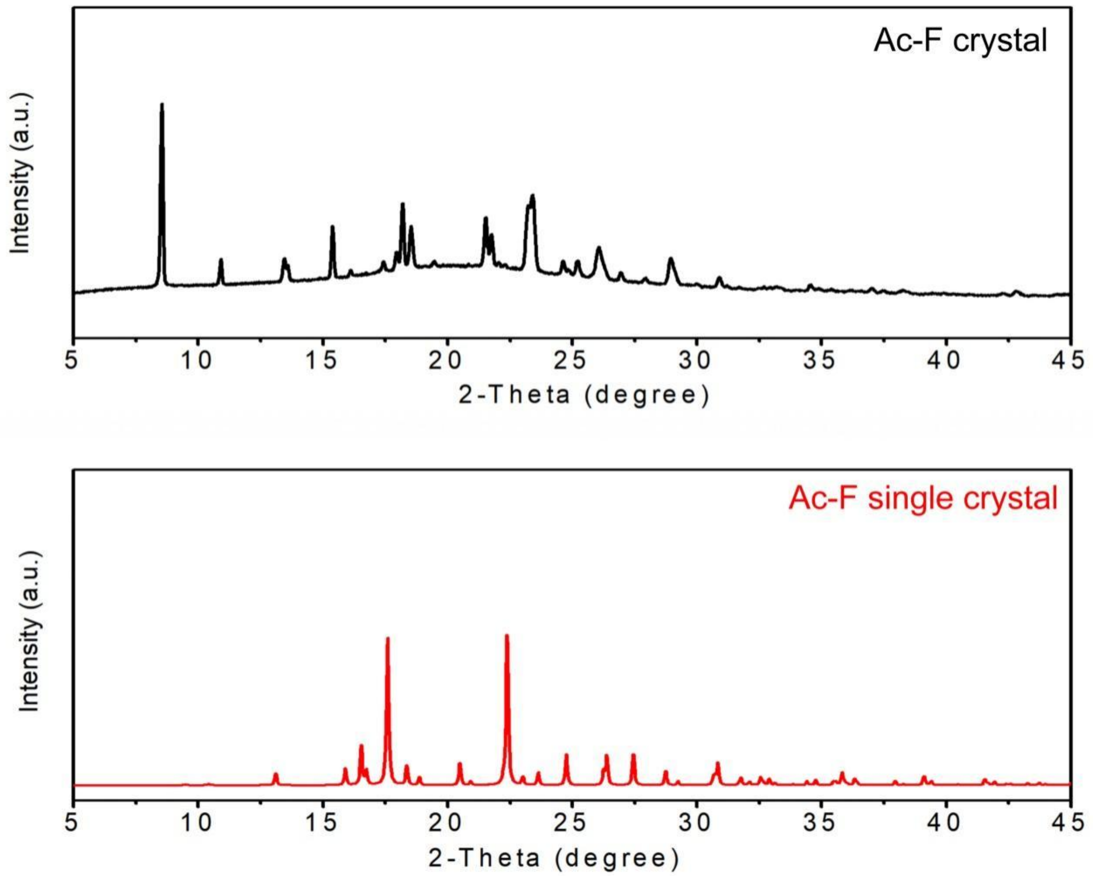

Fig. S20.

DSC curves (Upscan 2) of Ac–F *vs.* temperature with different heating rates (q_h) equal to the cooling rate (q_c), showing no change in the glass transition temperature when the cooling rate was over 10 K min^{-1}. The intersection of two dashed lines represents T_g.

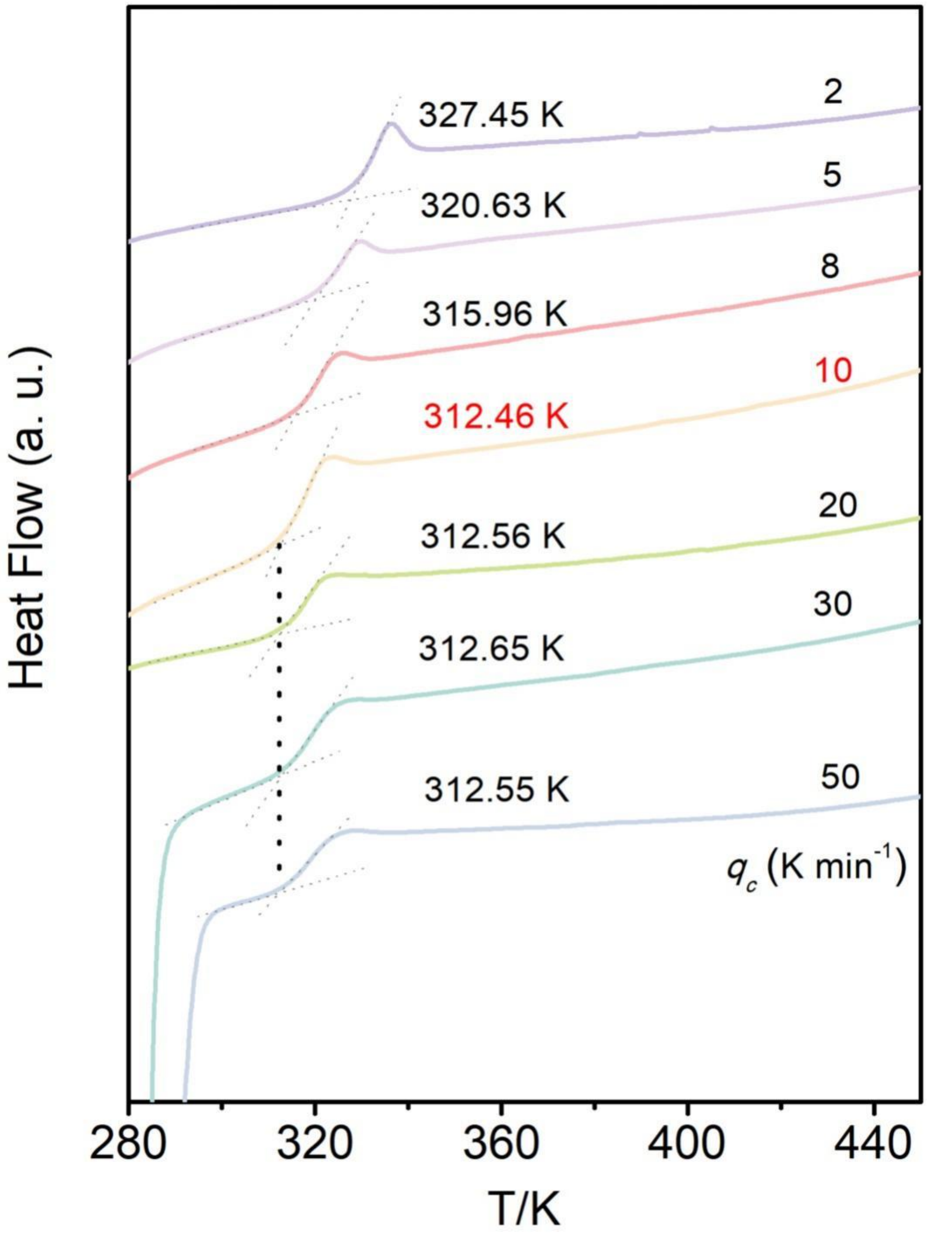

Fig. S21.

Thermogravimetric analysis and enthalpic responses of the typical Ac–amino acid glasses listed in Table S1. The intersection of two dashed lines in DSC curves represents T_g.

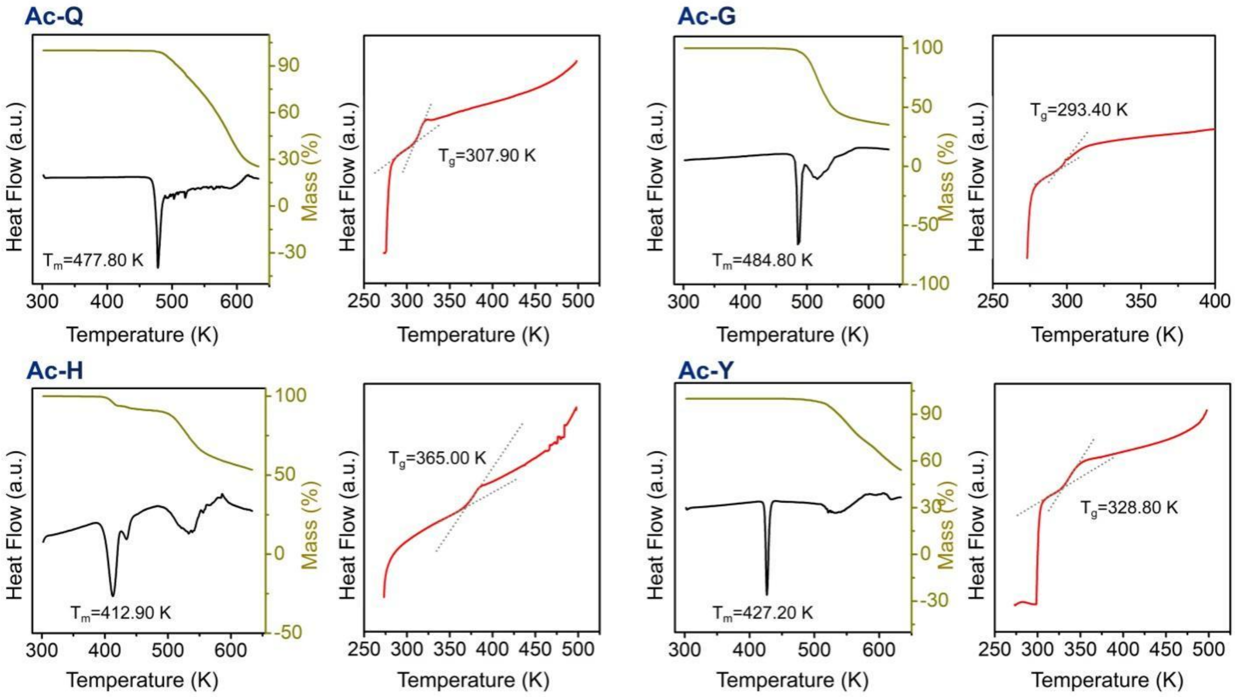

Fig. S22.

Thermogravimetric analysis and enthalpic responses of the typical Fmoc–amino acid glasses listed in Table S1. The intersection of two dashed lines represents T_g.

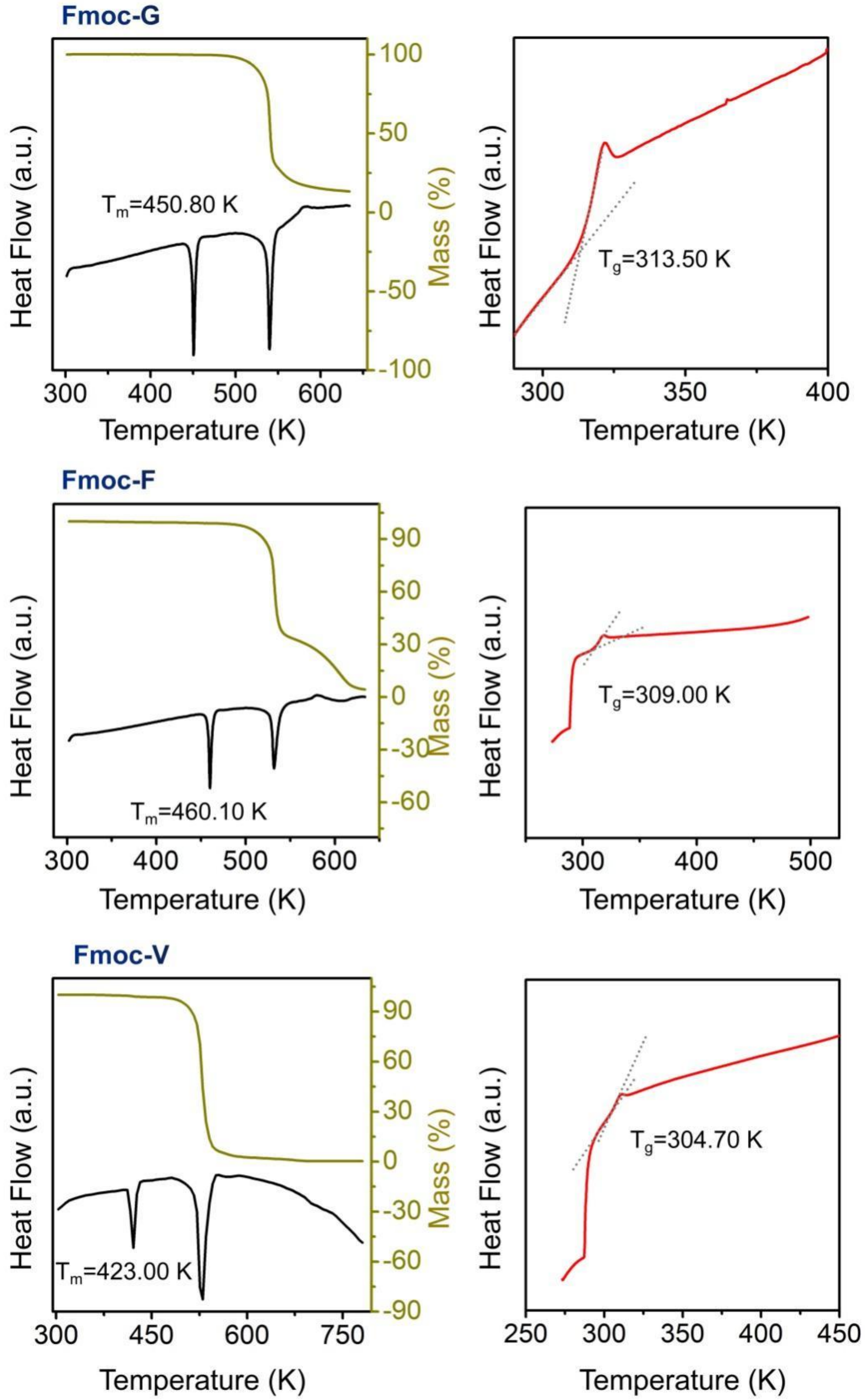

Fig. S23.

Thermogravimetric analysis and enthalpic responses of the typical Cbz–amino acid glasses listed in Table S1. The intersection of two dashed lines represents T_g.

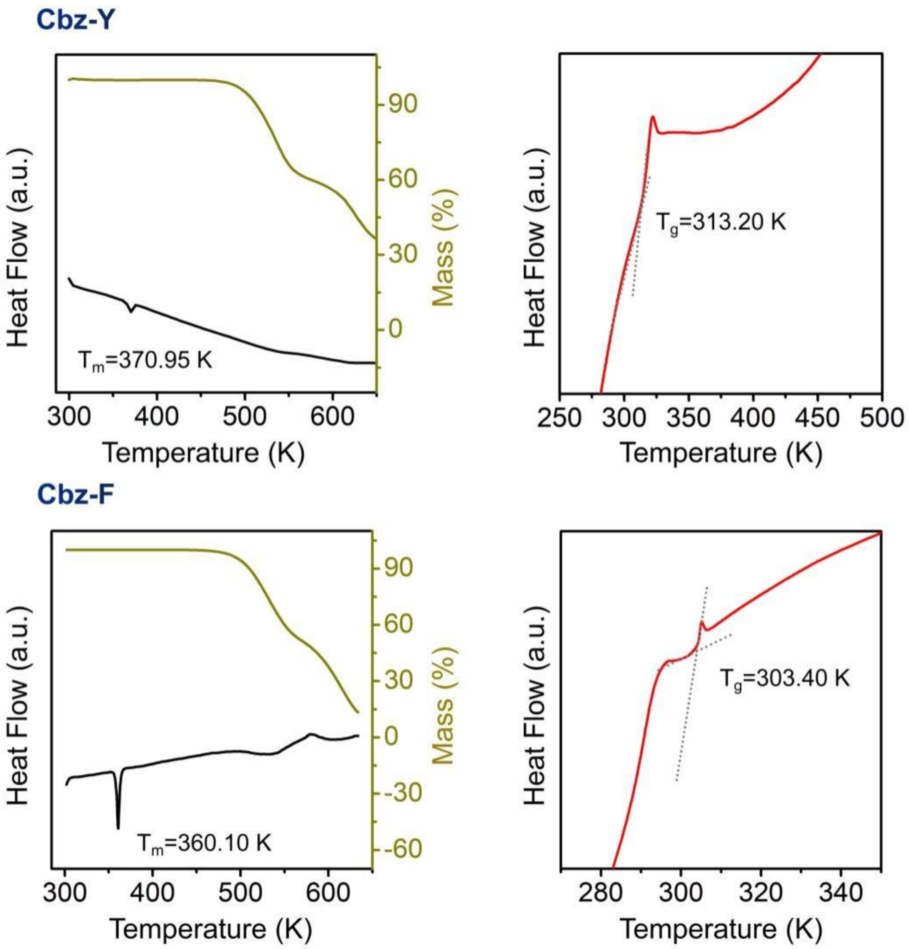

Fig. S24.

Thermogravimetric analysis and enthalpic responses of the typical peptide glasses listed in Table S1. The intersection of two dashed lines represents T_g.

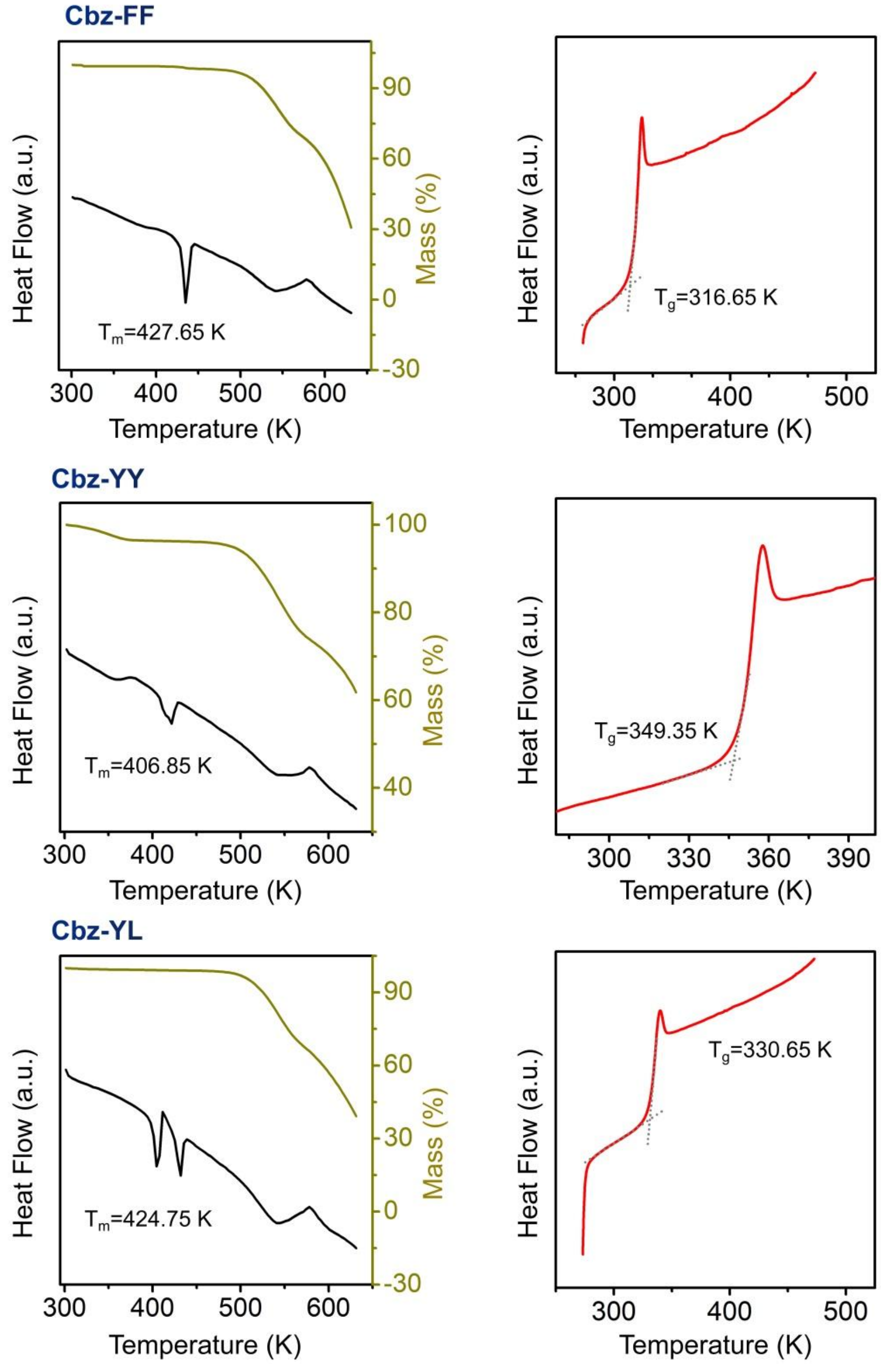

Fig. S25.

The fluorescence spectra of Ac–P glass, Fmoc–P glass, Ac–L glass, and Fmoc–L glass after mixing with equivalent of the Rhodamine B. The ratio of amino acid derivatives to dye is 1.0 mol: 0.05 mg.

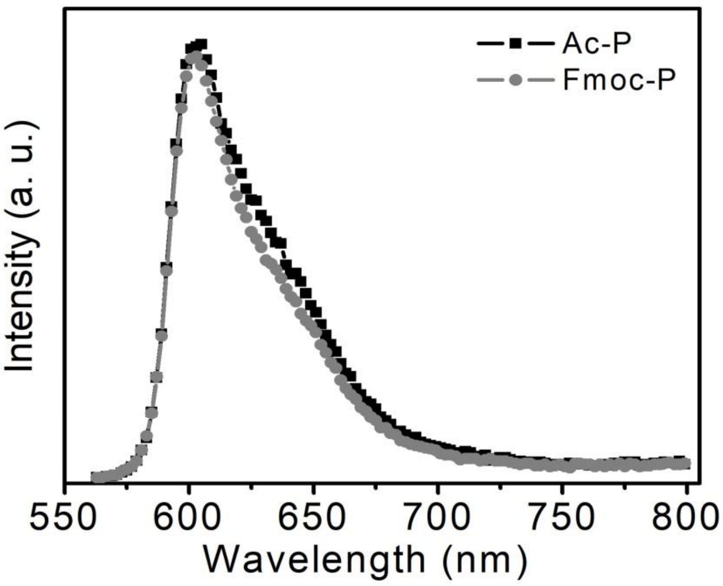

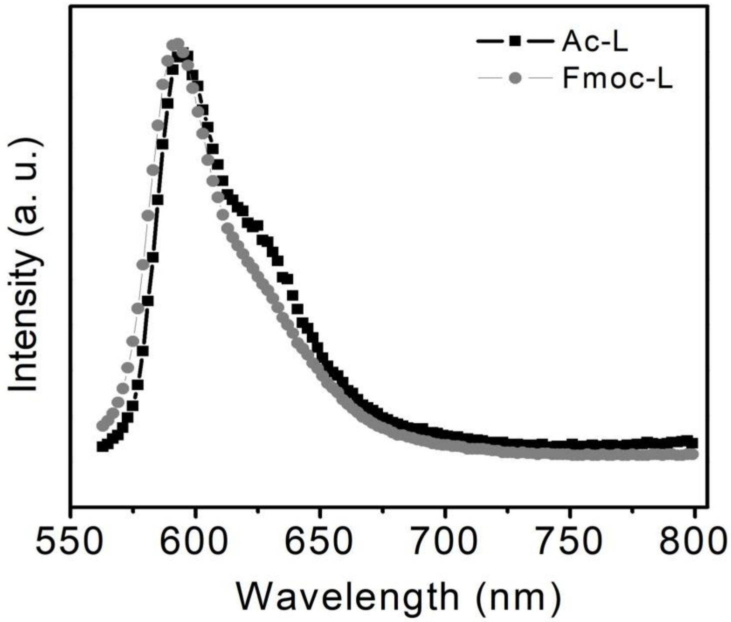

Fig. S26.

The XRD patterns of 3D–printed Ac–F glass, showing its amorphous nature.

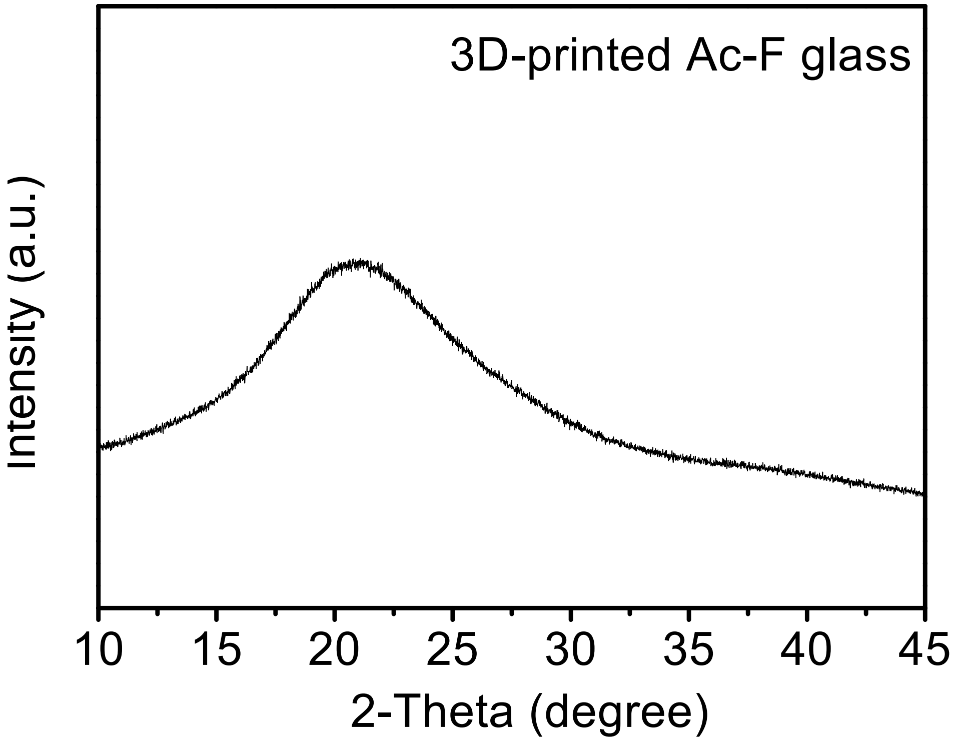

Fig. S27.

Viscosity curves of the Ac–F system as a function of temperature. The Ac–F powder was placed on the panel of a rheometer, and heated and melted (15 min at 473.15 K). Subsequently, the temperature was decreased to 273.15 K to obtain a viscous liquid. The process of increasing and decreasing the temperature was repeated three times. The strain was 0.1%, and the heating or cooling rates was ±10 K min^{-1}.

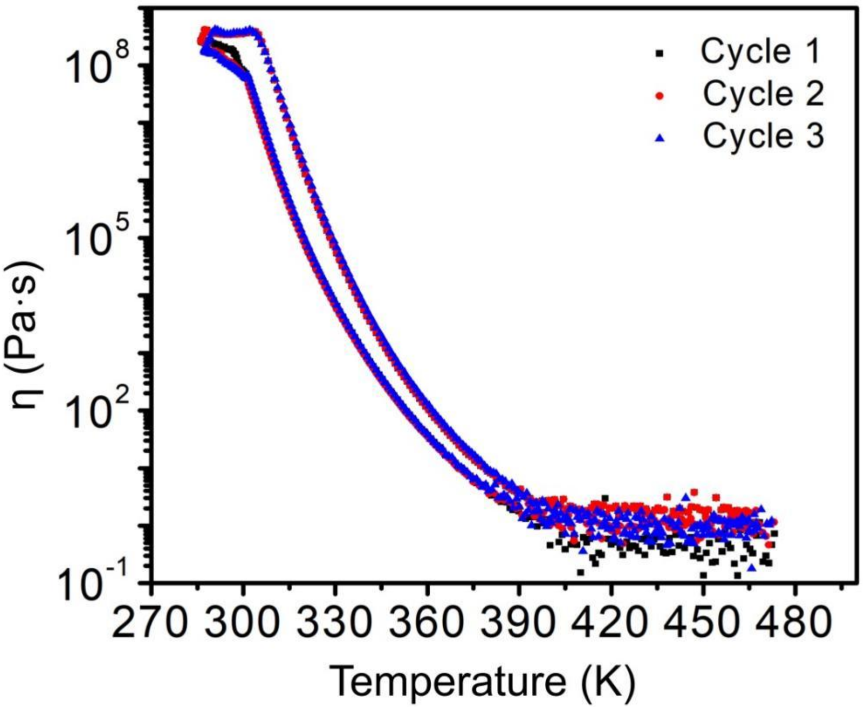

Fig. S28.

Degradation evaluation of amino acid and peptide glasses. Ac-F and Cbz-FFG glasses were exposed to proteinase K solution (0.1 mg mL^{-1}), simulated gastric fluid ($SGF_{[sp]}$) containing pepsin and simulated intestinal fluid ($SIF_{[sp]}$) containing pancreatin.

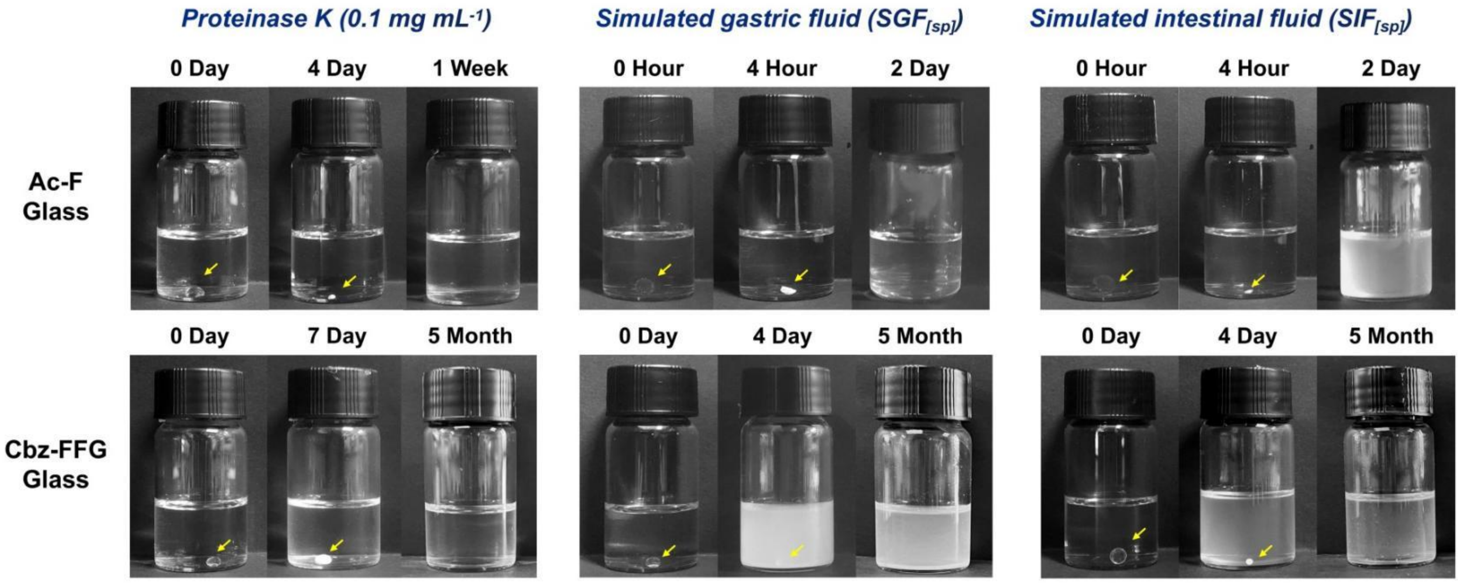

Fig. S29.

Mass spectrometry data for the degradation products of Cbz–FFG glass. The glass was incubated with proteinase K solution.

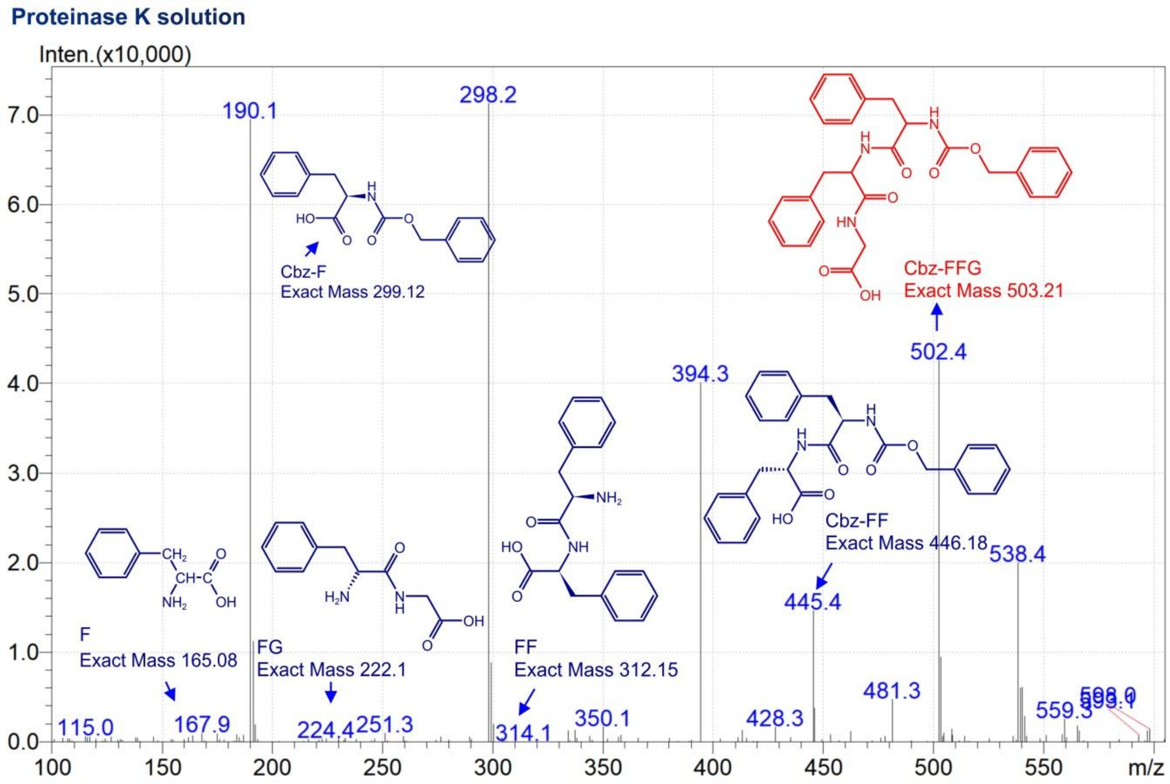

Fig. S30.

Mass spectrometry data for the degradation products of Cbz–FFG glass. The glass was incubated with simulated gastric fluid ($SGF_{[sp]}$) and simulated intestinal fluid ($SIF_{[sp]}$).

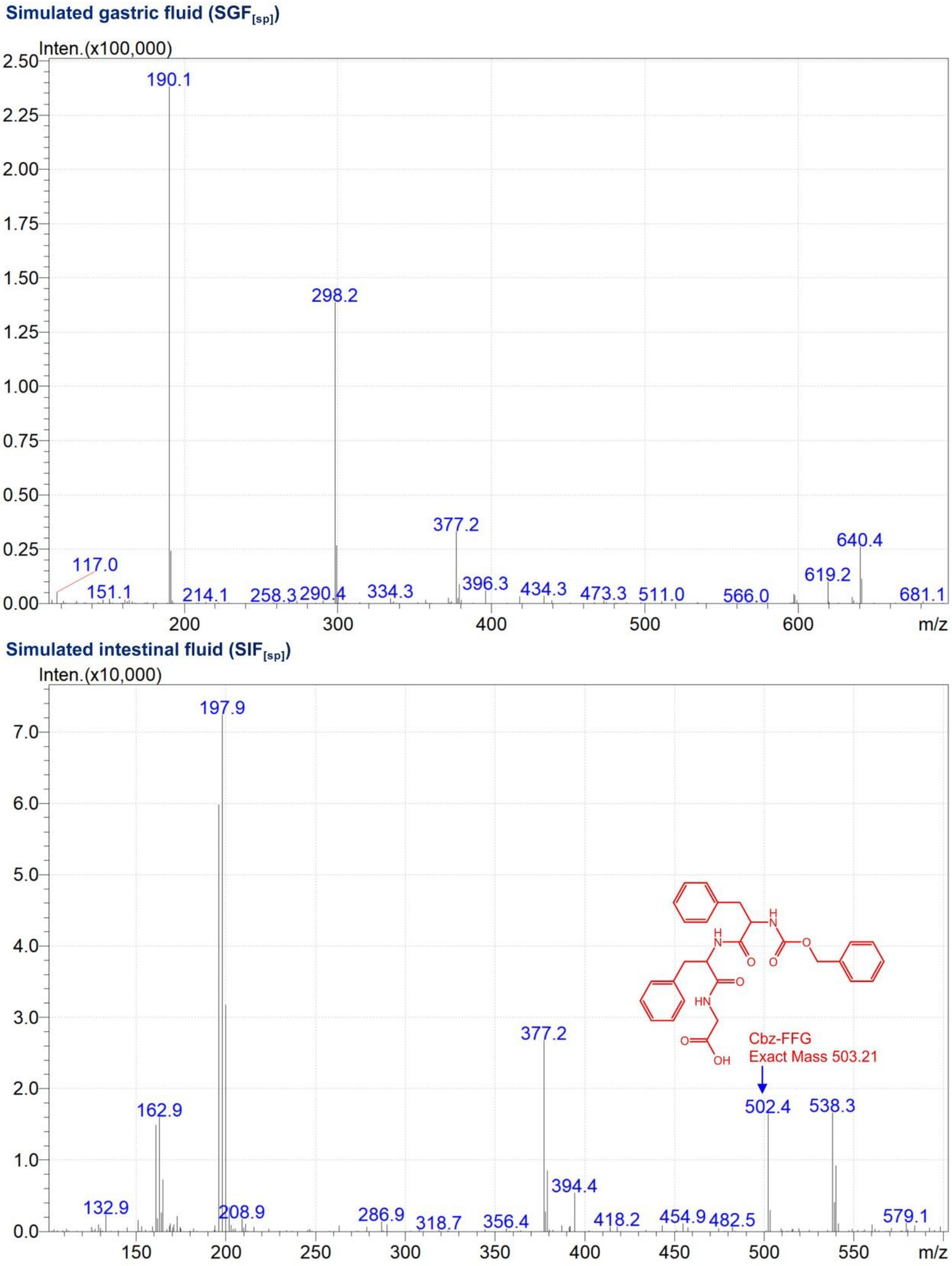

Fig. S31.

Pictures of Cbz–FFG glasses exposed to water over time, showing no discernible dissolution even after 10 days.

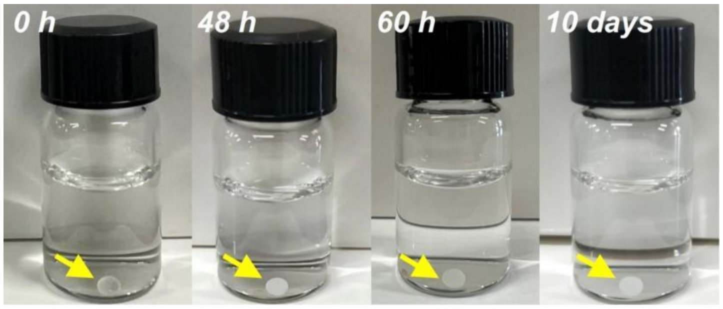

Fig. S32.

Histopathological analysis of the tissues in contact with the glass implants. The upper layer is skin tissue, and the lower layer is muscle tissue. The red arrows show inflammatory cell infiltration, and the black arrows show cuticle thickening.

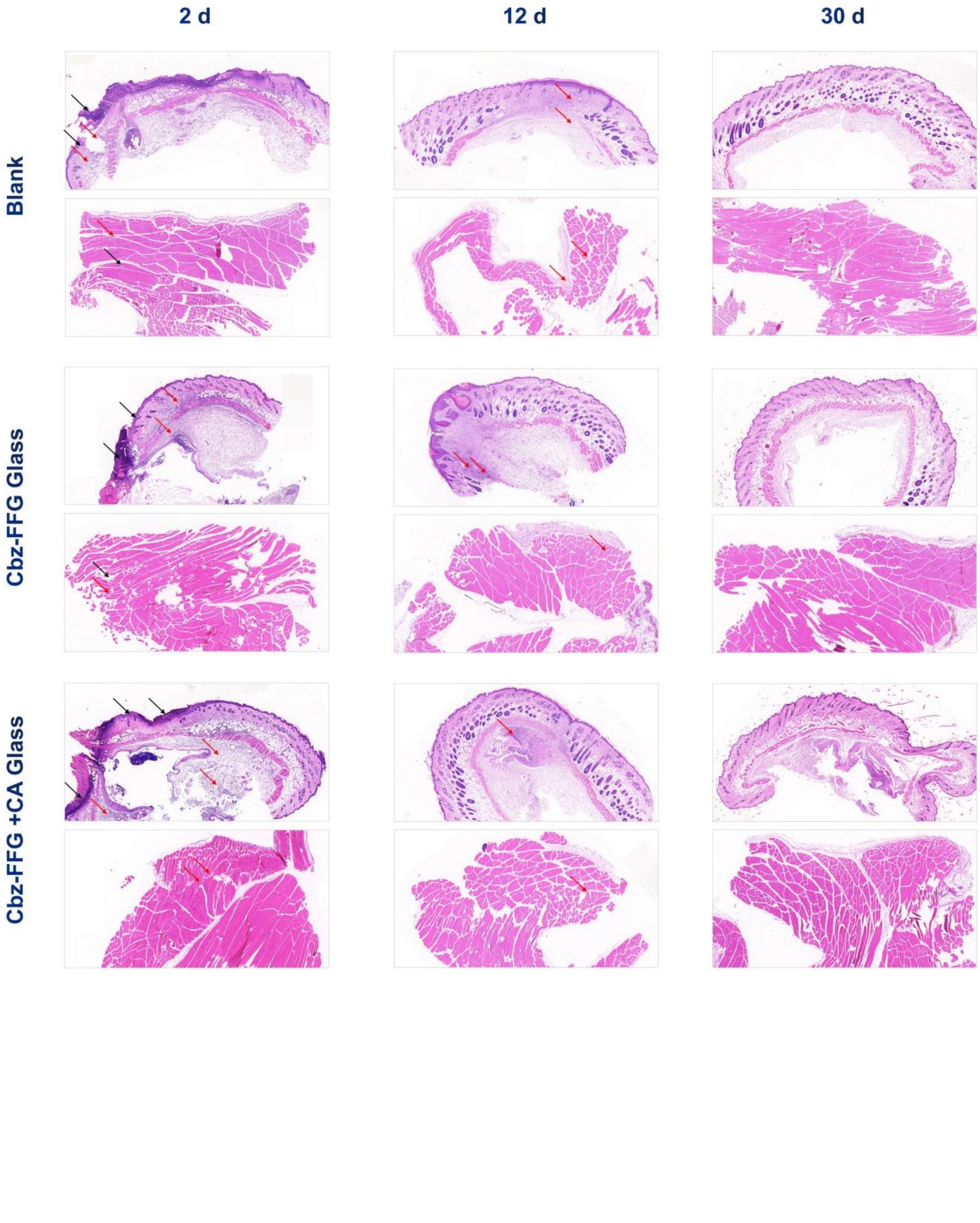

Supplement: Supplementary file 1 — Tables S1 to S3 Figs. S1 to S32 [file sciadv.add8105_sm.pdf]
